# Agricultural landscape simplification affects wild plant reproduction indirectly through herbivore-mediated changes in floral display

**DOI:** 10.1038/s41598-024-65352-2

**Published:** 2024-06-21

**Authors:** Hayley Schroeder, Annika Salzberg, Heather Grab, Shea Crowther, Casey Hale, Katja Poveda

**Affiliations:** 1 Department of Entomology, Cornell University, Ithaca, NY USA; 2 School of Integrative Plant Sciences, Cornell University, Ithaca, NY USA

**Keywords:** Landscape simplification, Plant–insect interactions, Plant traits, Reproductive fitness, *Barbarea vulgaris*, Pollination, Herbivory, Evolution, Ecology

## Abstract

As natural landscapes are modified and converted into simplified agricultural landscapes, the community composition and interactions of organisms persisting in these modified landscapes are altered. While many studies examine the consequences of these changing interactions for crops, few have evaluated the effects on wild plants. Here, we examine how pollinator and herbivore interactions affect reproductive success for wild resident and phytometer plants at sites along a landscape gradient ranging from natural to highly simplified. We tested the direct and indirect effects of landscape composition on plant traits and reproduction mediated by insect interactions. For phytometer plants exposed to herbivores, we found that greater landscape complexity corresponded with elevated herbivore damage, which reduced total flower production but increased individual flower size. Though larger flowers increased pollination, the reduction in flowers ultimately reduced plant reproductive success. Herbivory was also higher in complex landscapes for resident plants, but overall damage was low and therefore did not have a cascading effect on floral display and reproduction. This work highlights that landscape composition directly affects patterns of herbivory with cascading effects on pollination and wild plant reproduction. Further, the absence of an effect on reproduction for resident plants suggests that they may be adapted to their local insect community.

## Introduction

In recent decades, species richness has decreased precipitously in agroecosystems as a result of agricultural landscape simplification (hereafter referred to as landscape simplification)^1,2^. Declines in biodiversity result in reductions in ecosystem service provisioning^3^, with consequences for both crop productivity^4^ and ecosystem functioning^5,6^. In particular, documented declines in wild plant diversity in agroecosystems^7–10^ have important implications for agricultural production and ecosystem health due to the role semi-natural habitats play in promoting robust wild pollinator communities^11^ and mediating herbivorous insect damage^12,13^. While there is evidence that landscape composition is altering insect interactions with wild plants^14^, we currently lack a thorough understanding of the consequences of simultaneous shifts in mutualist and antagonist insect interactions for wild plant evolution.

Semi-natural habitats play a unique role in agroecosystems. They provide services such as food and nesting resources for pollinators^15,16^ and natural enemies^17,18^, while also functioning as a potential reservoir for herbivorous insects^19^ and source of weed establishment^20,21^ within a crop field. Therefore, the community composition and trait expression of a plant community are important in mediating the effects of wild plants on ecosystem function in agricultural systems^22–24^. Highly simplified landscapes are typically characterized by large areas of agriculture, with small patches of highly fragmented wild plant communities^25^ that experience regular disturbance^7^, chemical contamination^26^, and competition^27^. These environmental conditions are most favorable for plants that are competitive, rapid growing, and self-compatible^28^. Wild plants in agricultural landscapes additionally experience altered insect interactions due to landscape-mediated shifts in insect abundance and community composition, as well as the movement of insects between crops and natural plant communities^29^. As the insect community contains both antagonists and mutualists, their interactions could have important consequences for plant fitness, shaping the evolutionary trajectories of wild plants.

It is estimated that 87.5% of flowering plant species require animal pollination, which is primarily provided by insect pollinators^30–32^. Land use change, climate change, and increasing pesticide inputs are known drivers of the decline in pollinator richness and abundance worldwide^33^. Studies evaluating the consequences of pollinator losses have demonstrated intensified selection on floral traits^34,35^ and a reduction in plant diversity^36^. Highly simplified agricultural landscapes in particular are associated with reduced pollinator visitation^37^ and for non-crop plants in agricultural landscapes, proximity to mass blooming crops further impacts plant reproductive success^38–41^. By evaluating pollinator interactions with plants in semi-natural habitat patches along a gradient of increasing landscape simplification, we can make predictions about the relative contribution of pollinators to plant reproduction and ultimately how selection on plant traits may change with landscape composition.

Like pollinators, insect herbivore communities also change with increasing landscape simplification; however, the direction of their response is variable^42^. Generally, plants in simple landscapes experience elevated herbivore pressure by crop pests^43^, though this response can be species-specific^44^. While many studies evaluate how the surrounding landscape mediates herbivore abundance in crop systems^43,45^, dynamics for plants in neighboring semi-natural habitats are poorly understood. There is some evidence that herbivore populations can build up in crops and spill back into natural systems^46^, however spillover from managed to natural systems is likely underestimated due to a bias towards studies evaluating spillover in the opposite direction^29^. Two studies that specifically examined patterns of herbivory in wild plants found higher herbivory in complex systems and with increasing distance from crop relatives^47,48^.

In addition to this evidence that both pollinator and herbivore interactions with wild plants are changing with agricultural landscape simplification, there is a growing body of literature demonstrating that it is the combined effect of mutualist and antagonist insect interactions which mediates outcomes for wild plant fitness^49–51^. For example, damage by herbivores has been found to induce changes in floral traits with consequences for pollinator visitation^52,53^, resulting in either conflicting or reinforcing selection on plant traits^54^. However, despite evidence of landscape mediated changes in insect communities and the interactive effects of herbivores and pollinators on plant reproductive success, few studies have evaluated how variation in insect mutualist and antagonist interactions with wild plants across the landscape gradient affect wild plant reproduction^55^. Phytometer plants provide a useful tool for evaluating these interactions for wild plants by minimizing the contribution of adaptive trait variation. Because resident plant populations likely vary in their trait expression, potentially reflecting adaptation to their local environment, phytometer plants grown in a uniform environment from a single source plant allow for standardized measurements across many sites. Implementing pollinator and herbivore exclusion treatments with phytometer plants can help disentangle the individual contributions of pollinators and herbivores to plant reproductive success. Further, by examining both phytometer and resident plant populations we can begin to tease apart how landscape simplification mediates plant–insect interactions and whether local plant populations are adapting. A previous study examining the outcome of mutualist and antagonist insect interactions with phytometer plants in complex vs. simple landscapes found plant reproduction was affected interactively by pollinators and herbivores, but found only a weak contribution of the broader landscape^55^. We build on this work by constructing a structural equation modeling framework to evaluate the direct and indirect effects of landscape simplification, herbivory, and pollination on plant traits and reproductive success in the wild Brassicaceae species *Barbarea vulgaris*.

**Figure 1:**
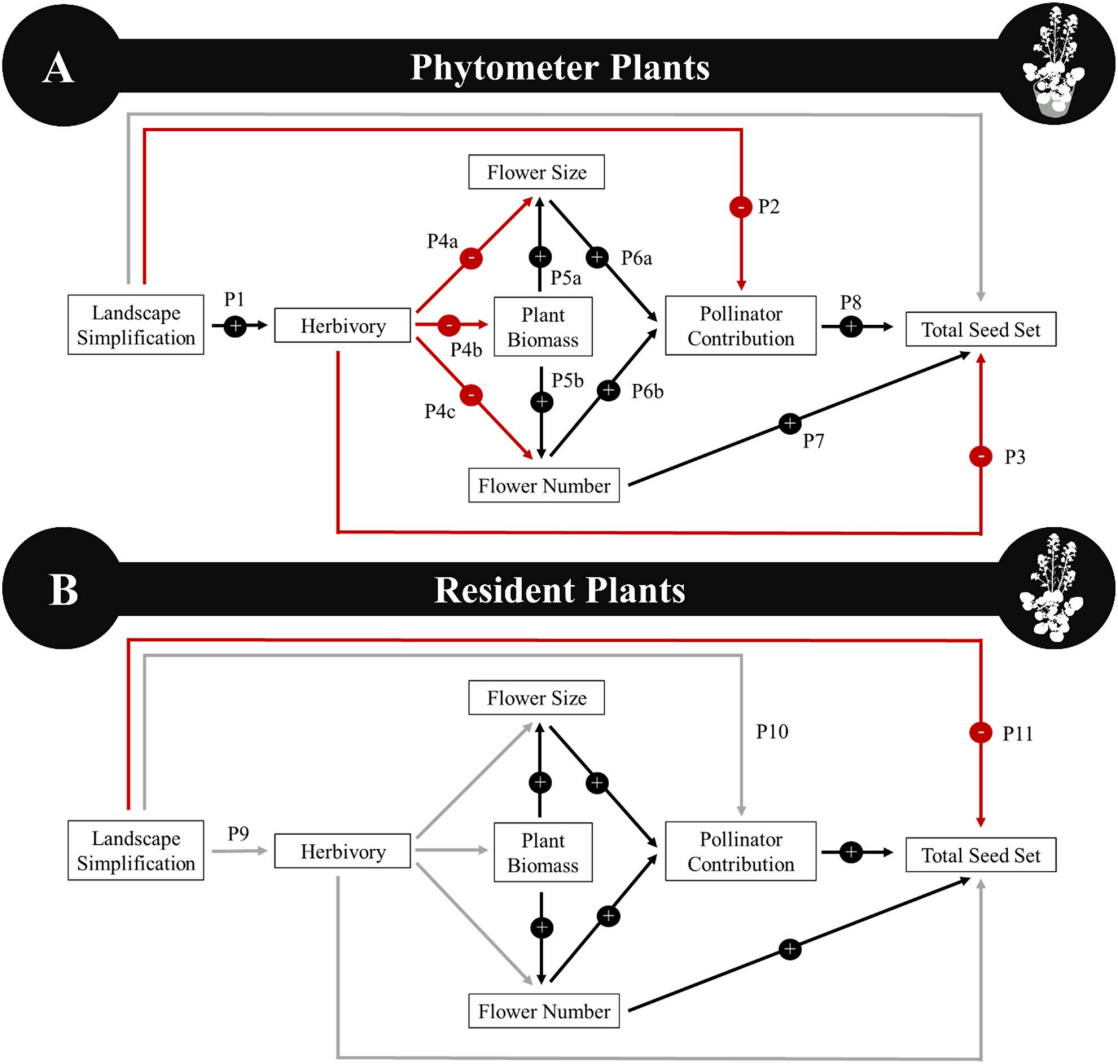
A schematic representation of our predictions for the direct and indirect paths by which landscape simplification can affect (**A**) phytometer and (**B**) resident plants. Landscape simplification represents increasing agricultural land cover in the surrounding landscape and pollinator contribution represents the difference in seed set between pollinator excluded and open inflorescences. Red lines indicate predicted negative relationships, black lines represent predicted positive relationships, and grey lines indicate predicted non-significant relationships. Numbers next to the lines refer to the predictions (P) that are referenced in the main text.

Previous work on *B. vulgaris* documented lower leaf consumption in herbivore bioassays (suggesting higher resistance) and smaller flower size (suggesting reduced pollinator visitation) for plants originating from simplified landscapes^56^. Therefore, we expect that for phytometer plants landscape simplification will increase herbivore damage (Fig. 1A; P1) and reduce insect pollination (Fig. 1A; P2). Based on the ecological cost hypothessis^57,58^, we expect that greater herbivory will reduce flower size (Fig. 1A; P4a), plant foliar biomass (Fig. 1A; P4b) and flower number (Fig. 1A, P4c), indirectly reducing seed set (Fig. 1a; P8) through reduced pollinator attraction (Fig. 1A; P6a, P6b)^53,59^. We expect flower number to be positively correlated with seed set by constraining the maximum possible reproductive output (Fig. 1A; P7). Because the phytometer plants will remain in their greenhouse pots and therefore be buffered from any variation in soil quality associated with landscape simplification, we do not expect there to be a direct effect of landscape on plant reproductive success. Ultimately, we predict that landscape simplification will indirectly reduce phytometer plant reproduction by reducing pollination and increasing herbivory, which will reduce plant reproductive success in agricultural landscapes.

If the resident plant population has adapted to compensate for changes in the insect community, we expect that there will be a weaker effect of landscape simplification on insect herbivory or pollination compared to the phytometer plants (Fig. 1B). *B. vulgaris* has been present in North America for over 200 years and documented in the Finger Lakes region for over 50 years^60^, and therefore has been established long enough for local adaptation to occur. Further, the landscape composition has been stable in this region for at least the last decade, potentially allowing for adaptation to the gradient of landscape simplification^56^. If the resident plants are locally adapted, we do not expect that the landscape will affect herbivory (Fig. 1B; P9) or pollination (Fig. 1B; P10). While we still predict that foliar biomass will positively influence floral display, which will increase pollination and seed set, we do not expect to find an indirect effect of landscape simplification mediated by herbivory. However, we do expect that landscape simplification will directly reduce plant reproduction due to the tradeoff between defense and reproduction (Fig. 1B; P11). We also predict that increasing landscape simplification will directly reduce plant reproductive success due to reduced soil quality resulting from intensive management (Fig. 1B; P11). However, it is also possible that synthetic fertilizer inputs in simplified landscapes result in increased soil quality, resulting in a positive direct effect of landscape simplification on plant reproductive success.

## Methods

### Study area

This study was conducted during the spring of 2020 in the Finger Lakes Region of upstate New York, USA. This region is characterized by a diversity of agricultural uses including row crops, vegetables, and fruit trees which vary in their management intensity, however in this study we examine the effects of agricultural land cover broadly rather than local management strategies. We identified 14 sites composed of agricultural field margins with a resident population of *B. vulgaris* and variation in habitat composition in the surrounding landscape (1.24–79.55% agriculture within a 1500 m radius). Sites were separated by a minimum of 2.5 km. We did not consider the effect of the adjacent crop given that the early bloom period of *B. vulgaris* (late April/early May) meant the experiment was completed prior to annual crop establishment. Two sites were eliminated from the analysis of the phytometer plants due to excessive desiccation of the plants at one site and prominent shade at the other which prevented the plants from blooming successfully. At each site we obtained informed consent from the landowner to establish experimental plots and ensured that all experimental protocols abided by relevant institutional and national guidelines.

### Study species

*Barbarea vulgaris*, known commonly as yellow rocket, was selected as a model species because it can be found growing in high abundance in a diversity of landscapes from predominantly natural to those dominated primarily by agriculture^56^. Although *B. vulgaris* is not native, it has been naturalized in North America for more than 200 years and has a rich community of pollinators and herbivores. While many species in heavily modified landscapes have high rates of autonomous self-pollination, *B. vulgaris* is self-compatible but depends on insect pollination for successful seed set. The flowers are visited mostly by wild bees and honey bees, but also by flies of the families Syrphidae and Anthomyiidae^61^. Near Ithaca, NY more than 25 species of herbivores have been found on *B. vulgaris* plants, with flea beetles of the genus *Phyllotreta* being the most common^60^.

### Resident focal plants

Within a resident population of *B. vulgaris* at each site we haphazardly selected 15 focal plants prior to bolting. Resident population densities were variable ranging from 0.02 to 5.15 plants/m^2^. We estimated plant density in the focal populations by running a 7.62 m transect through each population. The total number of plants was then counted within 5 circles with 1 m radii formed at randomly selected points along the transect. Pollinator exclusion bags were placed over one inflorescence on at least 5 plants per site.

### Phytometer plant plots

**Figure 2:**
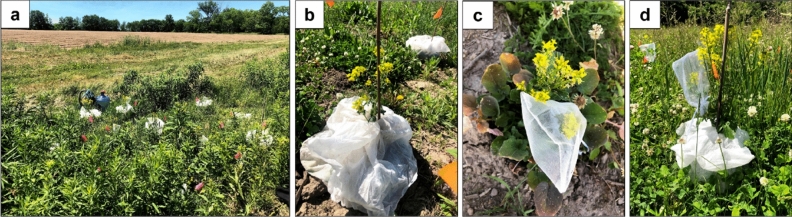
At each of the 14 study sites, 15 potted plants of Barbarea vulgaris were planted in patches within an agricultural landscape in a random array (**a**). For exclusion of all herbivorous insects, mesh bags were placed over the foliar rosette upon planting (**b**). For exclusion of pollinators, one inflorescence was covered with mesh bags (**c**). For a subset of plants with herbivore exclusion, one inflorescence was also bagged to exclude pollinators (**d**). Photos by Hayley Schroeder.

Phytometer plants were propagated in the greenhouse in January 2020 from a single parent plant sourced from an intermediate landscape (49.5% agriculture within a 1500 m radius). Because *B. vulgaris* is biennial, plants were transferred to a cooler 8 weeks post-germination to vernalize (4 °C 10 h photoperiod). Plants were then transferred to 3 L pots and moved to cold frames in early May to acclimate before being transferred to the field. Fifteen *B. vulgaris* plants were placed at each site at least five meters away from the resident population (Fig. 2a). Plants remained in their greenhouse pots to minimize the effect of variation in soil composition, and the pots were buried such that their rims were level with the surrounding soil. For the phytometer plants only, we placed herbivore exclusion bags (Breather; Palm Tree Packaging, Apopka, Florida, USA) over 7 randomly selected plants to isolate the contribution of herbivores to plant reproductive success (Fig. 2b). As a biennial, *B. vulgaris* germinates in the summer, growing throughout the fall and blooming the following spring. This means the resident plants had already experienced insect herbivory prior to the onset of the experiment, and therefore here we evaluate the combined effects of herbivores and pollinators for the resident plants. Once the plants bolted, we placed fine mesh pollinator exclusion bags over one inflorescence with buds on at least 5 plants per site, including both herbivore excluded and open plants for the phytometer plants (Fig. 2b–d). Pollinator exclusion bags were placed based on the timing of site visits (i.e. plants that already had most flowers open upon the first visit after bolting did not receive a pollinator exclusion bag) and the availability of inflorescences (i.e. plants with only one main inflorescence did not receive a pollinator exclusion bag), which ultimately resulted in an uneven number of replicates per treatment (see Supplemental Table S1 for full distribution of exclusion treatments). Additionally, one herbivore exclusion plant died prior to the conclusion of the experiment. To prevent desiccation, plants were watered when needed according to the weather. Resident and phytometer plant blooming periods did not overlap, with phytometer plants blooming immediately after the resident population.

### Landscape data

**Figure 3:**
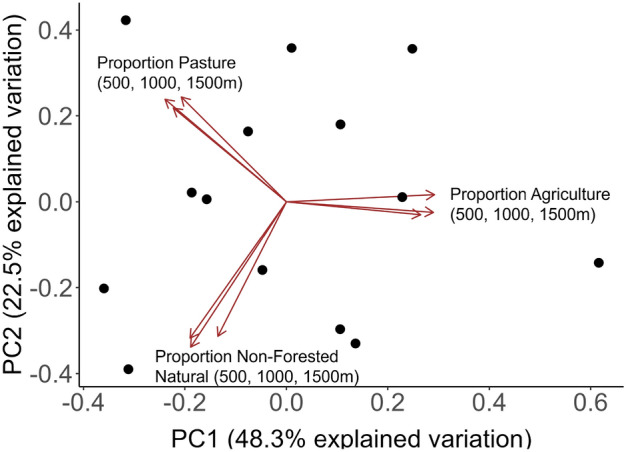
Results of a principal component analysis combining three landscape types (agriculture, pasture, and unforested natural land covers) found in previous work to be most predictive of Brassicaceae trait variation across three scales (500, 1000 and 1500 m). The first two principal components account for at least 70% of the variation in landscape composition. PC1 has positive and negative loadings reflecting agricultural land cover.

Land use composition at each site was evaluated using the 2020 National Agricultural Statistics Service Cropland Data Layer for New York (USDA 2020) in QGIS 3.18 (QGIS Development Team, QGIS Geographic Information System). Within a 500, 1000, and 1500 m radius of each experimental plot, we calculated the proportion of land cover categorized as agriculture, pasture, urban development, natural open, and natural forested land area. Given the high number of landscape variables and scales, we retained three land cover classifications found in previous work to be most predictive of Brassicaceae trait variation (agriculture, non-forest natural land cover, and pasture)^56^ and then conducted a principal component analysis to produce PC1 and PC2 values that summarized the relative landscape composition of each source site (Fig. 3). The PC1 values represent increasing agricultural land cover, explaining 48.3% of the variation in landscape composition, and were used in all further analyses.

### Herbivory

To assess herbivore damage, we visually estimated the percent leaf tissue removed on 5 haphazardly selected leaves per plant and also recorded an estimate of whole plant damage. After the initial damage assessment beginning May 20th, we measured plant foliar damage every other week across the experiment for the phytometer plants open to insect herbivory for a total of four visits concluding on July 8th. Foliar herbivory on the resident plants was only measured once during the first damage assessment when the focal plants were first selected because the rosettes senesced once the plants bolted, whereas phytometer plants sustained their foliar rosettes throughout the experiment. Foliar damage on herbivore-excluded phytometer plants was only recorded once at the end of the experiment to minimize exposing the plants to herbivores by opening the bags. To identify the dominant herbivore in this system, we also collected herbivores from each plant by vacuuming the rosette leaves and buds for 10 s. Herbivores were collected from the phytometer plants once a week (5 times in total) throughout the experiment while resident plants were vacuumed only once at the beginning of the experiment due to the senescence of the rosette. Collected insects were euthanized with dry ice, grouped by site and date, and identified to order in the lab. Flea beetles were counted separately and identified to species as they were the most dominant herbivore in previous work^60^.

### Pollinator visitation

Pollinator visitation and community data was collected to provide ecological context for broader patterns documented in the path analysis. For the resident plant populations, we observed each focal plant with open accessible blooms at the time of each visit for five minutes, recording all insect visitors classified by morphotype. Each contact an insect made with flower reproductive structures was considered a unique visit. Depending on the length of the bloom period, visitation was recorded for each resident plant during 1–3 observation periods. Because of their proximity to one another, all the phytometer plants with open accessible blooms at a site were observed together during a single 20-min observation period, resulting in 1–6 observation periods depending on the duration of bloom for each plant. To evaluate variation in the pollinator community, we collected specimens for identification by conducting a 10 min transect walk in a neighboring *B. vulgaris* patch (to avoid damaging the focal plants) and collecting all visitors using sweep netting. Two collection surveys were conducted at each site between 9:00 and 16:00 on sunny days with temperatures above 15 °C. Insects were euthanized with dry ice and brought back to the lab to identify to the highest classification possible using published revisions (Supplemental Table S5) and online keys (Discoverlife.org, treehoppers.insectmuseum.org) and confirmed through reference materials maintained in the Cornell University Insect Collection. All collected specimens are deposited into the Cornell University Insect Collection (Accession Numbers 67025-671200).

### Floral traits

Once the plants bolted, we collected one of the first open flowers, removing all the petals and fixing them to a piece of paper with tape. Petal length and width were measured to the nearest hundredth mm using an Olympus SZX10 stereomicroscope and CellSens measurement software (Olympus Corp. Tokyo, Japan). The length of the longest petal was used to represent flower size. The total flowers produced was calculated (using the reproductive success data below) as the summation of all fertile and aborted pods across both bagged and unbagged portions of a plant.

### Plant reproductive success

To evaluate plant reproductive success we counted all aborted and fertile seed pods on each focal plant, separating branches that were bagged (pollinator exclusion) or unbagged. A seed pod was classified as fertile if there was evidence that at least one seed was forming. The resident focal plants were harvested once the seed pods were fully developed and then allowed to further ripen in paper bags. Phytometer plants were brought in from the field once the plants had completed blooming and were harvested once the seed pods were fully dry. For both the resident and phytometer plants, bagged and unbagged branches were harvested separately. For the phytometer plants, we obtained the dry mass of all remaining foliar plant tissue. This data was not available for the resident plant populations as very few plants maintained a rosette after bolting. We estimated pollinator contribution to plant reproduction by subtracting the proportion of fertile seed pods on bagged branches from the proportion of fertile seed pods on the unbagged portion of the plant. *B. vulgaris* is self-compatible and produces some seeds in the absence of pollinators, therefore by subtracting the proportion seed set of the bagged from the open branches, we are able account for the selfing potential of the plant and estimate the contribution of pollinators to seed set. To estimate plant reproduction success given the bagging treatment, we used the proportion seed set on the open portion of the plant to estimate the theoretical seed set of the bagged branches and added this estimate to the total fertile seed pods count.

### Statistical analysis

All statistical analyses were conducted in R, version 4.3.1^62^. We built structural equation models (SEMs) to evaluate the causal factors influencing plant reproduction using the R package ‘lavaan’^63^. SEMs are a useful tool for simultaneously testing multiple complex relationships and statistically evaluating indirect pathways. Using this framework, we built separate SEMs for resident plants and phytometer plants with and without herbivory. In all models we included landscape composition, herbivore damage, flower size, flower number, and pollinator contribution as direct or indirect predictors of total seed set. Pollinator contribution to seed set was selected rather than visitation rate as the variable to represent pollination because it represents the overall outcome of pollinator interactions rather than a snapshot in time and accounts for the selfing potential of the plant. The package ‘lavaan’ is also unable to support the zero inflated structure of the visitation data. For phytometer plants we additionally included dried leaf biomass as a predictor of flower size and number and response to herbivory. Because the resident plant rosettes senesced upon bloom, dried leaf biomass was not used as a predictor for resident plants. Damage estimates were log-transformed to improve model fit and plant biomass, flower number, flower size, and total seed set were mean-centered and scaled to unit variance. All models were estimated by full information maximum likelihood. Because the SEM package ‘lavaan’ does not allow for the incorporation of random effects, we ran individual linear models for each path with and without site as a random factor and found comparable beta coefficients (Supplementary Tables S2–4). To be conservative, we have shifted the significance threshold for individual paths from 0.05 to 0.01 to account for the non-independence of plants within a site.

We evaluated diversity, richness, and evenness of the pollinator community across the landscape gradient using the R package ‘vegan’^64^. Separate analyses were performed for bees (Hymenoptera), hoverflies (Syrphidae), and for all insects collected. Insects that were damaged during collection or storage (N = 29) and male bees (N = 10) were removed from the analysis due to difficulty in identification. Community analyses for bees and hoverflies were evaluated at the species level, while family was used for analysis of all insects collected as this was the highest level of classification with complete data.

## Results

### Insect community

#### Vacuum collected herbivores

Across all sampling periods we collected 3375 insects, with Coleoptera being the most abundant order present. Of the 1712 Coleoptera collected, 1495 were flea beetles, with *Phyllotreta striolata* being the most abundant (N = 866), followed by *Phyllotreta cruciferae* (N = 394), and *Psylliodes punctulata* (N = 235). Hemipterans were the next most abundant order (N = 677) followed by Diptera (N = 506), though flies collected were likely visiting the flowers or resting on the plant. The final herbivores collected in appreciable abundance were gastropods (N = 94).

#### Collected pollinators and floral visitation

We collected 710 insects while sweep-netting *B. vulgaris* flowers. Of these insects, there were 270 bees representing at least 58 species and 154 hoverflies representing 16 species. The remaining insects represented 47 families across 7 orders (See Supplemental Table S5 for complete list). Landscape composition was not a significant predictor of diversity, richness, or evenness for bees, hoverflies, or insects overall across either sampling period, therefore they were not included in any further analysis (Supplemental Fig. S1; Supplemental Table S6). Overall, we documented 1198 flower visits to the phytometer plants during 8,140 min of observation and 605 visits to resident plants over 1170 min (Supplemental Table S7). Bees in the genus *Lasioglossum* were the most abundant visitors, accounting for 40% of all visits. See Supplemental Tables S8 and S9 for detailed visitation results for phytometer and resident plants.

### General results

**Figure 4:**
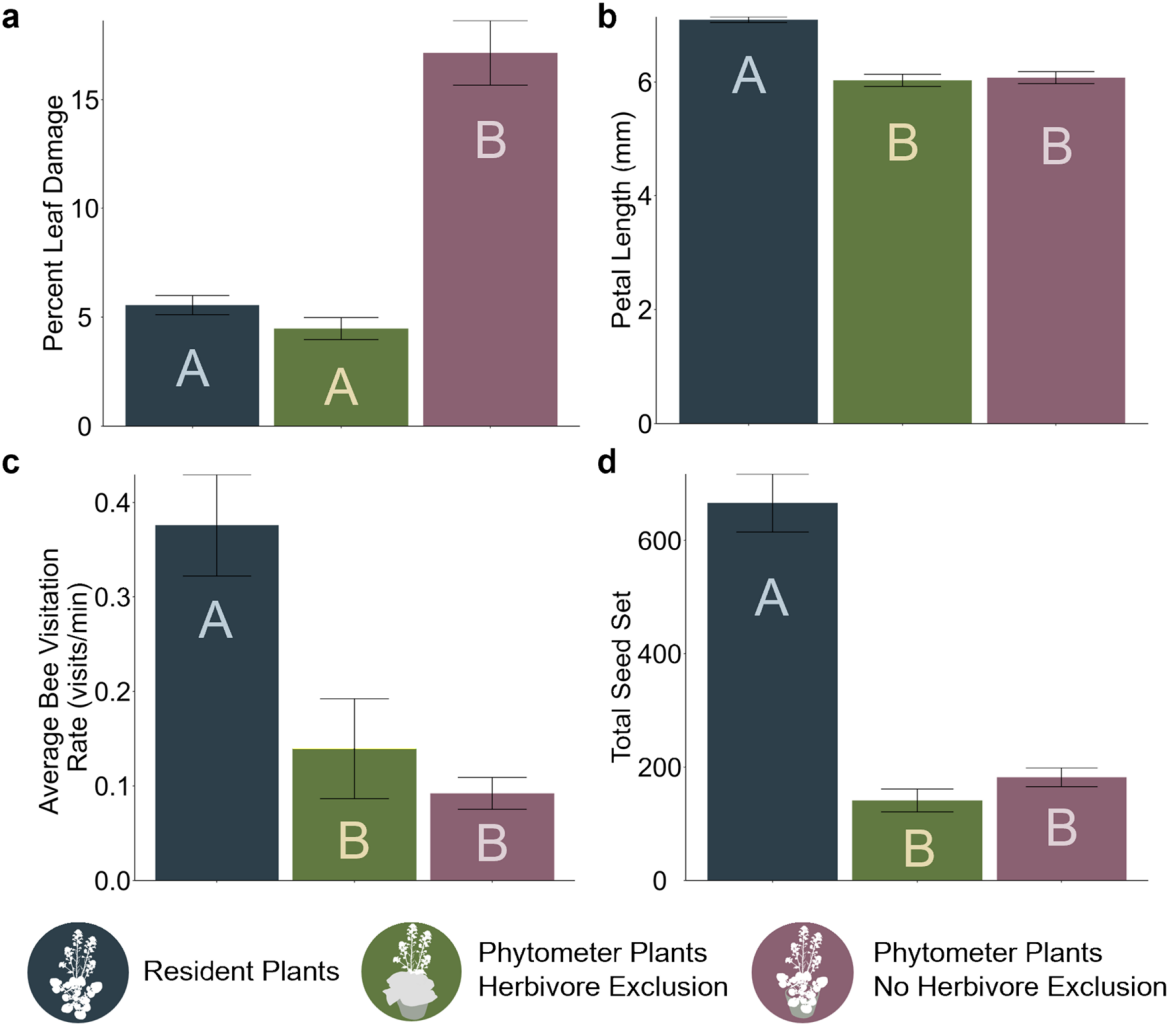
Mean values for percent leaf damage (**a**), petal length (**b**), average bee visitation rate (**c**), and total seed set (**d**) across all resident plants, phytometer plants with herbivore exclusion, and phytometer plants open to herbivory. Letters indicate statistically significant differences between means in a pairwise comparison using the Tukey method. Error bars represent one standard error.

The herbivore exclusion treatment was successful in reducing foliar herbivory, with herbivore excluded phytometer plants receiving significantly less damage than those open to insect herbivory (*F*_2,371_ = 64.12, *P* < 0.0001; Fig. 4a). Resident plant populations produced larger flowers than both herbivore-excluded and open phytometer plants (*F*_2,263_ = 46.84, *P* < 0.0001; Fig. 4b). Additionally, resident plants experienced a significantly higher bee visitation rate (*F*_2,277_ = 6.77, *P* = 0.001; Fig. 4c) and overall seed production (*F*_2,336_ = 38.59, *P* < 0.0001; Fig. 4d) than both herbivore-excluded and open phytometer plants.

### Structural equation models

#### Phytometer plants open to herbivory

**Figure 5:**
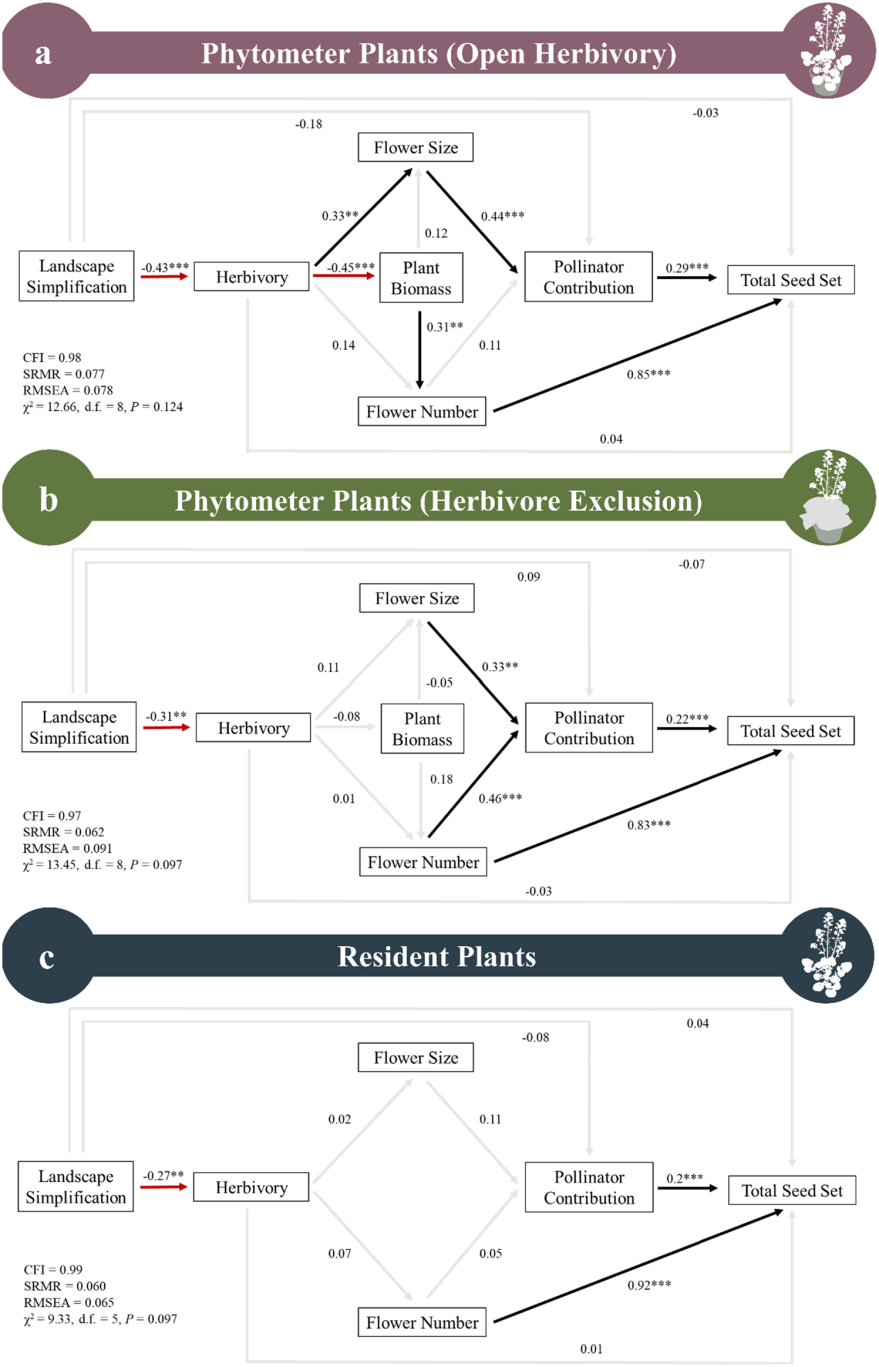
Structural equation model exploring the direct and indirect relationships between landscape simplification, herbivory, plant foliar biomass, floral display (flower size and total flower number), pollinator contribution (the difference in the proportion seed set between bagged and open inflorescences), and total seed set for (**a**) phytometer plants open to insect herbivory, (**b**) Phytometer plants with herbivore exclusion, and (**c**) resident plants. Plant foliar biomass is not included for resident plants due to rosette senescence after bolting. Black arrows represent significant positive relationships, red arrows represent significant negative relationships, and grey arrows indicate non-significant relationships. To account for the non-independence of the plants within a site, the significance threshold for individual paths is 0.01. Values adjacent to the lines are standardized path coefficients. ***P < 0.001, **P < 0.01, *P < 0.05, ˙*P* < 0.10.

While there was no direct effect of landscape simplification on the total seed set, we found two significant indirect pathways (Fig. 5a). In one pathway, landscape simplification negatively affected seed set through reduced herbivory, which resulted in reduced flower size and therefore reduced pollinator contribution (z = − 1.97, *P* = 0.049). However, in the second pathway, landscape simplification resulted in a positive effect on plant fitness because reduced herbivory increased plant foliar biomass, resulting in more flowers and thus greater seed set (z = 2.06, *P* = 0.039). When comparing the standardized path coefficients for both indirect pathways, the positive effect on fitness is 2.83 times greater than the negative effect, suggesting that the overall outcome of landscape simplification on plant fitness is positive.

#### Phytometer plants with herbivore exclusion

Landscape simplification did not directly affect total seed set, and there were no indirect pathways in which the landscape mediated plant fitness. While the herbivore exclusion treatment reduced plant damage by 73.89% compared to open treatments (F_1,177_ = 58.17, *P* < 0.0001), landscape simplification still reduced herbivory for herbivore excluded plants (Fig. 5b). However, this reduced herbivory did not significantly affect plant foliar biomass, flower size, or flower number. Greater flower size and number increased pollinator contribution which increased total seed set, and flower number also directly increased total seed set.

#### Resident plants

For the focal resident plants (Fig. 5c), landscape simplification also reduced herbivory. However, herbivory had no effect on flower size or flower number. Additionally, flower size and number did not have a significant effect on pollination. Greater pollinator contribution and flower number both directly increased the total seed set. Landscape simplification did not significantly affect the total seed set and there also were no significant indirect pathways by which the landscape affected seed production.

## Discussion

The unprecedented scale at which natural landscapes have been converted to agriculture across the last two centuries has spurred countless studies examining the consequences for biodiversity, ecosystem services, and crop productivity^65–67^. While there is significant evidence that this land use change alters community composition, it remains largely unexplored how changing interactions may be altering the evolutionary trajectories for organisms persisting in heavily modified landscapes. Recently, two studies found altered selection on plant reproductive and defensive traits associated with proximity to monoculture crops^41,49^. Here, we build on this work by demonstrating the indirect pathways through which landscape simplification may affect wild plant reproduction. Because we evaluate a single generation in this study we use plant reproduction as a proxy for fitness potential, as these two metrics are closely linked^68,69^, however this method captures only female reproduction and does not account for the viability of the following generation. Here we found that in simplified landscapes, phytometer plants open to insect herbivory experienced reduced herbivore damage which resulted in a cascading reduction in individual flower size and increase in overall flower number. Although smaller flower size reduced pollinator contribution, increased flower number had a stronger effect on plant reproduction such that landscape simplification had an overall positive effect on seed set. This work highlights the importance of examining the interplay of mutualist and antagonist interactions when investigating the consequences of landscape simplification for ecological dynamics in wild plants.

The documented reduction in herbivory in simple landscapes runs counter to our hypothesis that landscape simplification would increase insect herbivory. In previous work informing this hypothesis, *Trichoplusia ni* caterpillars consumed less leaf area from *B. vulgaris* plants originating from simplified landscapes, suggesting higher resistance in these landscapes^56^. However, lepidopteran herbivores were found in low abundances in the field, and therefore are likely not the most effective predictor of resistance in the field. The reduction in damage in simple landscapes may indicate that insect populations over all are reduced in simplified landscapes due to intensive management and pesticide applications^70^*.* The reduction in damage for all plant types in simplified landscapes is consistent with other studies that have found reduced herbivory on wild plants close to monoculture crops^48^ and in landscapes with little non-crop habitat^47^. Flea beetles were found to be the most dominant herbivore feeding on *B. vulgaris*, and in other work the highest abundances of flea beetles were documented in landscapes with greater pasture land cover (in this study complex landscapes have higher proportion of pasture)^71^. Given evidence that flea beetles prefer to overwinter outside agricultural fields^72^, the varied habitat available in more complex landscapes may provide more suitable overwintering locations and thus result in larger populations early in the spring.

For open phytometer plants, elevated herbivory documented in complex landscapes resulted in contrasting cascading effects on floral traits, pollination, and reproduction. Increased herbivore damage resulted in reduced total flower number but increased individual flower size. While increased herbivory in complex landscapes may divert energy from plant growth, resulting in lower foliar biomass and reduced flower number, plants may compensate by increasing floral attractiveness^73^. However, this result runs counter to the frequently documented reduction in flower size as an indirect result of foliar herbivory^58,59,74^. Though larger flower size increased the total seed set indirectly through increased insect pollination, ultimately the reduction in overall flower production still had a stronger negative effect on plant reproduction in complex landscapes. This indirect positive association with larger flower size mediated by herbivores in complex landscapes aligns with previous work documenting larger flowers in *B. vulgaris* plants originating from complex compared to simple landscapes^56^ and provides evidence that the combined interactions of pollinators and herbivores may alter selection on flower size across the landscape gradient. Overall, flower number was consistently the strongest predictor of total seed set in all SEMs, highlighting the importance of flower production in mediating both potential reproductive success and attractiveness to pollinators. Thus, by indirectly affecting flower production, herbivores appear to play a significant role in mediating plant reproductive success. This finding contributes to a body of literature demonstrating the importance of insect herbivores as agents of selection on plant traits^75–77^.

This connection between herbivory and floral display and pollination is absent for both herbivore-excluded phytometer plants and resident plants, both of which experienced significantly reduced herbivory overall compared to the open phytometer plants. Because the indirect effect of landscape simplification on plant reproduction for open phytometer plants was driven by herbivore mediated changes in floral display, without sufficient damage to alter floral phenotypes there was no indirect effect of landscape simplification on plant reproduction. The fact that overall herbivore pressure was significantly reduced in resident plant populations could suggest that the resident plants are adapted to their local herbivore community. However, as greenhouse-raised plants, the phytometer plants may have been more susceptible or nutritious to herbivores. Additionally, because the phenologies of the resident and phytometer plants were not perfectly synchronous, further work is necessary to disentangle how much the disparity in herbivore damage can be attributed to local adaptation vs. phenological variation.

While the phytometer plants demonstrated changes in insect interactions in the absence of local adaptation, the resident populations of *B. vulgaris* provide insight into the capacity of the plant community to compensate for these changing interactions. Though we still found a reduction in herbivore damage associated with increasing landscape simplification for the resident plant populations, the amount of damage the plants received overall was significantly reduced across all sites. Without high levels of insect herbivory, there were no cascading effects of damage on floral display and therefore pollination. A lack of direct or indirect effects of landscape simplification on resident plant’s reproductive success indicates that they have similar reproductive success across all landscapes and may suggest that the resident plants have adapted to their local environments. This aligns with previous work documenting differential trait expression in floral display and herbivore resistance associated with landscape simplification for *B. vulgaris*^56^. However, this study is limited in the conclusions that can be drawn about adaptation occurring in existing plant populations as the data collected on resident populations is observational. Future research should specifically test for differential selection on plant traits mediated by insects across the landscape gradient. Additionally, while a recent study similarly found no direct effects of landscape composition on pollination or reproductive success in phytometer plants^55^, we highlight here the potential to expose novel patterns by evaluating both direct and indirect relationships.

Though we expected landscape simplification to negatively affect the pollinator community visiting *B. vulgaris*^78^, we found no effect on the diversity, richness, or evenness of pollinators visiting *B. vulgaris*. Importantly, this reflects the pollinator community specifically visiting *B. vulgaris* rather than the community present in the landscape broadly. The most abundant floral visitors were bees in the genus *Lasioglossum,* which have been found to be among the most resilient clades to landscape simplification^33^. The absence of an effect of landscape simplification on the pollinator community is consistent with the results of our SEMs, which indicated no direct effect of landscape on pollinator contribution, but rather an indirect effect as a result of herbivore mediated changes in floral display. This finding is also consistent with another study which found no effect of landscape complexity on pollinator richness, and a significant effect of herbivores in mediating floral attractiveness^55^. Separately, a meta-analysis evaluating bee responses to human disturbance documented declines in pollinator richness, but only for landscapes experiencing extreme habitat loss (> 95% disturbance in the surrounding landscape)^79^. Therefore, it is possible that the range of landscape simplification evaluated here was not sufficient to detect pollinator declines, particularly for resilient, generalist species. Additionally, *B. vulgaris* is pollinated by generalist pollinators, which are likely to be more resilient to landscape modification than more sensitive specialist pollinators^80^.

To date, studies documenting the consequences of landscape simplification focus largely on patterns in cropping systems, leaving a dearth of information regarding dynamics for wild plants persisting within modified landscapes. Here we demonstrate that landscape simplification may indirectly affect wild plant reproduction through herbivore mediated changes in floral display. This highlights the importance of examining the combined outcome of both mutualist and antagonist interactions when evaluating the consequences of landscape simplification for plant populations. Given that plant trait expression can reflect adaptation to the local community of interactors, understanding the consequences of changing insect interactions for plant reproduction can inform predictions about the evolutionary trajectories for wild plants that have the potential to reinforce changes in the insect community.

### Supplementary Information

Supplementary Information.

## Data Availability

The datasets generated during and/or analyzed during the current study are available in the Dryad repository, [10.5061/dryad.905qfttt6].

## References

[1] Tilman D, Cassman KG, Matson PA, Naylor R, Polasky S (2002). Agricultural sustainability and intensive production practices. Nature.

[2] Tilman D (2001). Forecasting agriculturally driven global environmental change. Science.

[3] Mace GM, Norris K, Fitter AH (2012). Biodiversity and ecosystem services: A multilayered relationship. Trends Ecol. Evol..

[4] Carvalheiro LG (2011). Natural and within-farmland biodiversity enhances crop productivity. Ecol. Lett..

[5] Isbell F (2015). Biodiversity increases the resistance of ecosystem productivity to climate extremes. Nature.

[6] Isbell F (2011). High plant diversity is needed to maintain ecosystem services. Nature.

[7] Roschewitz I, Gabriel D, Tscharntke T, Thies C (2005). The effects of landscape complexity on arable weed species diversity in organic and conventional farming. J. Appl. Ecol..

[8] Hall RM (2020). Vegetation management intensity and landscape diversity alter plant species richness, functional traits and community composition across European vineyards. Agric. Syst..

[9] Clough Y (2014). Density of insect-pollinated grassland plants decreases with increasing surrounding land-use intensity. Ecol. Lett..

[10] Richner N, Holderegger R, Linder HP, Walter T (2015). Reviewing change in the arable flora of Europe: A meta-analysis. Weed Res..

[11] Kammerer MA, Biddinger DJ, Rajotte EG, Mortensen DA (2016). Local plant diversity across multiple habitats supports a diverse wild bee community in Pennsylvania apple orchards. Environ. Entomol..

[12] Crowther LI, Wilson K, Wilby A (2023). The impact of field margins on biological pest control: A meta-analysis. BioControl.

[13] Balzan MV, Bocci G, Moonen A-C (2016). Landscape complexity and field margin vegetation diversity enhance natural enemies and reduce herbivory by Lepidoptera pests on tomato crop. BioControl.

[14] Schroeder H, Grab H, Kessler A, Poveda K (2021). Human-mediated land use change drives intraspecific plant trait variation. Front. Plant Sci..

[15] Purvis EEN, Meehan ML, Lindo Z (2020). Agricultural field margins provide food and nesting resources to bumble bees (*Bombus* spp., Hymenoptera: Apidae) in Southwestern Ontario, Canada. Insect Conserv. Divers..

[16] Aviron S, Berry T, Leroy D, Savary G, Alignier A (2023). Wild plants in hedgerows and weeds in crop fields are important floral resources for wild flower-visiting insects, independently of the presence of intercrops. Agric. Ecosyst. Environ..

[17] Karamaouna F (2022). Selected flowering plants as a habitat for pollinators and natural enemies in field margins of a watermelon crop—implications for crop yield. Int. J. Pest Manag..

[18] Mkenda PA (2019). Field margin vegetation in tropical african bean systems harbours diverse natural enemies for biological pest control in adjacent crops. Sustainability.

[19] Pollier A, Guillomo L, Tricault Y, Plantegenest M, Bischoff A (2018). Effects of spontaneous field margin vegetation on the regulation of herbivores in two winter crops. Basic Appl. Ecol..

[20] Visscher AM (2023). Drivers of growth and establishment of the invasive plant *Rumex acetosella* within Andean fallow systems. Agric. Ecosyst. Environ..

[21] Guerra JG, Cabello F, Fernández-Quintanilla C, Peña JM, Dorado J (2022). How weed management influence plant community composition, taxonomic diversity and crop yield: A long-term study in a Mediterranean vineyard. Agric. Ecosyst. Environ..

[22] Schuldt A (2019). Multiple plant diversity components drive consumer communities across ecosystems. Nat. Commun..

[23] Beugnon R, Eisenhauer N, Bohan DA, Dumbrell AJ (2019). Chapter five - Plant functional trait identity and diversity effects on soil meso- and macrofauna in an experimental grassland. Advances in Ecological Research.

[24] Storkey J (2013). Using functional traits to quantify the value of plant communities to invertebrate ecosystem service providers in arable landscapes. J. Ecol..

[25] Steffan-Dewenter I, Tscharntke T (1999). Effects of habitat isolation on pollinator communities and seed set. Oecologia.

[26] Botías C, David A, Hill EM, Goulson D (2016). Contamination of wild plants near neonicotinoid seed-treated crops, and implications for non-target insects. Sci. Total Environ..

[27] Wassmuth BE, Stoll P, Tscharntke T, Thies C (2009). Spatial aggregation facilitates coexistence and diversity of wild plant species in field margins. Perspect. Plant Ecol. Evol. Syst..

[28] Navas M-L (2012). Trait-based approaches to unravelling the assembly of weed communities and their impact on agro-ecosystem functioning. Weed Res..

[29] Blitzer EJ (2012). Spillover of functionally important organisms between managed and natural habitats. Agric. Ecosyst. Environ..

[30] Ollerton J, Winfree R, Tarrant S (2011). How many flowering plants are pollinated by animals?. Oikos.

[31] Potts SG (2016). Safeguarding pollinators and their values to human well-being. Nature.

[32] Vanbergen AJ (2013). Threats to an ecosystem service: Pressures on pollinators. Front. Ecol. Environ..

[33] Grab H (2019). Agriculturally dominated landscapes reduce bee phylogenetic diversity and pollination services. Science.

[34] Panique H, Caruso CM (2020). Simulated pollinator declines intensify selection on floral traits that facilitate selfing and outcrossing in Impatiens capensis. Am. J. Bot..

[35] Hossack GC, Caruso CM (2023). Simulated pollinator decline has similar effects on seed production of female and hermaphrodite Lobelia siphilitica, but different effects on selection on floral traits. Am. J. Bot..

[36] Lundgren R, Totland Ø, Lázaro A (2016). Experimental simulation of pollinator decline causes community-wide reductions in seedling diversity and abundance. Ecology.

[37] Chatterjee A, Chatterjee S, Smith B, Basu P (2020). Determinants of bee visitation in an economically important vegetable crop along an agricultural intensification gradient. Proc. Zool. Soc..

[38] Van Reeth C, Michel N, Bockstaller C, Caro G (2019). Influences of oilseed rape area and aggregation on pollinator abundance and reproductive success of a co-flowering wild plant. Agric. Ecosyst. Environ..

[39] Holzschuh A, Dormann CF, Tscharntke T, Steffan-Dewenter I (2011). Expansion of mass-flowering crops leads to transient pollinator dilution and reduced wild plant pollination. Proc. R. Soc. B Biol. Sci..

[40] Montero-Castaño A, Ortiz-Sánchez FJ, Vilà M (2016). Mass flowering crops in a patchy agricultural landscape can reduce bee abundance in adjacent shrublands. Agric. Ecosyst. Environ..

[41] Qiu Y (2023). Proximity to oilseed rape fields affects plant pollination and pollinator-mediated selection on a co-flowering plant on the Tibetan Plateau. Evol. Appl..

[42] Karp DS (2018). Crop pests and predators exhibit inconsistent responses to surrounding landscape composition. Proc. Natl. Acad. Sci..

[43] Rand TA, Waters DK, Blodgett SL, Knodel JJ, Harris MO (2014). Increased area of a highly suitable host crop increases herbivore pressure in intensified agricultural landscapes. Agric. Ecosyst. Environ..

[44] Dong Z (2020). Landscape agricultural simplification correlates positively with the spatial distribution of a specialist yet negatively with a generalist pest. Sci. Rep..

[45] Poveda K, Martínez E, Kersch-Becker MF, Bonilla MA, Tscharntke T (2012). Landscape simplification and altitude affect biodiversity, herbivory and Andean potato yield. J. Appl. Ecol..

[46] Moxley C (2017). A major subtropical fruit pest accumulates in crop fields and spills over to a wild host. Agric. Ecosyst. Environ..

[47] Clough Y, Kruess A, Tscharntke T (2007). Local and landscape factors in differently managed arable fields affect the insect herbivore community of a non-crop plant species. J. Appl. Ecol..

[48] Chamberlain SA, Whitney KD, Rudgers JA (2013). Proximity to agriculture alters abundance and community composition of wild sunflower mutualists and antagonists. Ecosphere.

[49] Mitchell N, Chamberlain SA, Whitney KD (2021). Proximity to crop relatives determines some patterns of natural selection in a wild sunflower. Evol. Appl..

[50] Hoffmeister M, Wittköpper N, Junker RR (2016). Herbivore-induced changes in flower scent and morphology affect the structure of flower–visitor networks but not plant reproduction. Oikos.

[51] Soper Gorden NL, Adler LS (2018). Consequences of multiple flower–insect interactions for subsequent plant–insect interactions and plant reproduction. Am. J. Bot..

[52] Rusman Q, Poelman EH, Nowrin F, Polder G, Lucas-Barbosa D (2019). Floral plasticity: Herbivore-species-specific-induced changes in flower traits with contrasting effects on pollinator visitation. Plant Cell Environ..

[53] Strauss SY (1997). Floral characters link herbivores, pollinators, and plant fitness. Ecology.

[54] Sletvold N, Moritz KK, Ågren J (2015). Additive effects of pollinators and herbivores result in both conflicting and reinforcing selection on floral traits. Ecology.

[55] Grass I, Bohle V, Tscharntke T, Westphal C (2018). How plant reproductive success is determined by the interplay of antagonists and mutualists. Ecosphere.

[56] Schroeder H, Grab H, Poveda K (2023). Phenotypic clines in herbivore resistance and reproductive traits in wild plants along an agricultural gradient. PLOS ONE.

[57] Strauss SY, Siemens DH, Decher MB, Mitchell-Olds T (1999). Ecological costs of plant resistance to herbivores in the currency of pollination. Evolution.

[58] Lehtilä K, Strauss SY (1999). Effects of foliar herbivory on male and female reproductive traits of wild radish, Raphanus Raphanistrum. Ecology.

[59] Strauss SY, Conner JK, Rush SL (1996). Foliar herbivory affects floral characters and plant attractiveness to pollinators: Implications for male and female plant fitness. Am. Nat..

[60] Root RB, Tahvanainen JO (1969). Role of winter cress, *Barbarea vulgaris*, as a temporal host in the seasonal development of the crucifer fauna1. Ann. Entomol. Soc. Am..

[61] Dailey TB, Scott PE (2006). Spring nectar sources for solitary bees and flies in a landscape of deciduous forest and agricultural fields: Production, variability, and consumption1. J. Torrey Bot. Soc..

[62] R Core Team. *R: A Language and Environment for Statistical Computing* (R Foundation for Statistical Computing, 2023). <https://www.R-project.org/>.

[63] Rosseel Y (2012). lavaan: An R package for structural equation modeling. J. Stat. Softw..

[64] Oksanen, J. *et al.**The vegan package* (2009).

[65] Connelly H, Poveda K, Loeb G (2015). Landscape simplification decreases wild bee pollination services to strawberry. Agric. Ecosyst. Environ..

[66] Gámez-Virués S (2015). Landscape simplification filters species traits and drives biotic homogenization. Nat. Commun..

[67] Nelson KS, Burchfield EK (2021). Landscape complexity and US crop production. Nat. Food.

[68] Younginger BS, Sirová D, Cruzan MB, Ballhorn DJ (2017). Is biomass a reliable estimate of plant fitness?. Appl. Plant Sci..

[69] Primack RB, Kang H (1989). Measuring fitness and natural selection in wild plant populations. Annu. Rev. Ecol. Syst..

[70] Hallmann CA (2017). More than 75 percent decline over 27 years in total flying insect biomass in protected areas. PLOS ONE.

[71] Perez-Alvarez R, Nault BA, Poveda K (2018). Contrasting effects of landscape composition on crop yield mediated by specialist herbivores. Ecol. Appl..

[72] Andersen CL, Hazzard R, Van Driesche R, Mangan FX (2005). Overwintering and seasonal patterns of feeding and reproduction in *Phyllotreta cruciferae* (Coleoptera: Chrysomelidae) in the Northeastern United States. Environ. Entomol..

[73] Schiestl FP, Kirk H, Bigler L, Cozzolino S, Desurmont GA (2014). Herbivory and floral signaling: Phenotypic plasticity and tradeoffs between reproduction and indirect defense. New Phytol..

[74] Jacobsen DJ, Raguso RA (2018). Lingering effects of herbivory and plant defenses on pollinators. Curr. Biol..

[75] Santangelo JS, Thompson KA, Johnson MTJ (2019). Herbivores and plant defences affect selection on plant reproductive traits more strongly than pollinators. J. Evol. Biol..

[76] Gómez JM (2003). Herbivory reduces the strength of pollinator-mediated selection in the mediterranean herb *Erysimum mediohispanicum*: Consequences for plant specialization. Am. Nat..

[77] Parachnowitsch AL, Caruso CM (2008). Predispersal seed herbivores, not pollinators, exert selection on floral traits via female fitness. Ecology.

[78] Holzschuh A (2016). Mass-flowering crops dilute pollinator abundance in agricultural landscapes across Europe. Ecol. Lett..

[79] Winfree R, Aguilar R, Vázquez DP, LeBuhn G, Aizen MA (2009). A meta-analysis of bees’ responses to anthropogenic disturbance. Ecology.

[80] Redhead JW (2018). Potential landscape-scale pollinator networks across Great Britain: Structure, stability and influence of agricultural land cover. Ecol. Lett..

