# Supplementary material for: Agricultural landscape simplification affects wild plant reproduction indirectly through herbivore-mediated changes in floral display
Source: Sci Rep. 2024 Jun 21;14:14293. doi: 10.1038/s41598-024-65352-2 (PMC11192729; doi:10.1038/s41598-024-65352-2)
Supplement: Supplementary file 1 — Supplementary Information. [file 41598_2024_65352_MOESM1_ESM.docx]

**Supplementary Information**

**Agricultural landscape simplification affects wild plant reproduction indirectly through herbivore mediated changes in floral display**

Hayley Schroeder*, Annika Salzberg, Heather Grab, Shea Crowther, Casey Hale, and Katja Poveda

*Corresponding author

E-mail: [hayleyadair37@gmail.com](mailto:hayleyadair37@gmail.com)

**Supplementary Figure S1**

**Supplementary Tables S1, S2, S3, S4, S5, S6 & S7**

**Supplementary Figure S1.** Species diversity, richness, and evenness for bees and hoverflies collected from resident *B. vulgaris* plants

**Supplementary Table S1.** Number of replicates in each treatment combination for each site.

**Supplementary Table S2.**  Results of simple linear models and linear mixed-effects models with site included as a random effect for phytometer plants open to herbivory.

**Supplementary Table S3.** Results of simple linear models and linear mixed-effects models with site included as a random effect for herbivore excluded phytometer plants.

**Supplementary Table S4.** Results of simple linear models and linear mixed-effects models with site included as a random effect for resident plants.

**Supplementary Table S5.** Insects collected on flowers of *Barbarea vulgaris* by sweep netting resident plants

**Supplementary Table S6.** Results of linear models evaluating agricultural landscape simplification as a predictor of three metrics of insect community composition

**Supplementary Table S7.** Pollinator visitation by species/morpho-group identified “on-the-wing” visiting *B. vulgaris* phytometer and resident plants.

**Supplementary Table S8.** Results of zero inflated negative binomial generalized mixed linear models evaluating pollinator visitation to phytometer plants

**Supplementary Table S9.** Results of zero inflated negative binomial generalized mixed linear models evaluating pollinator visitation to resident plants


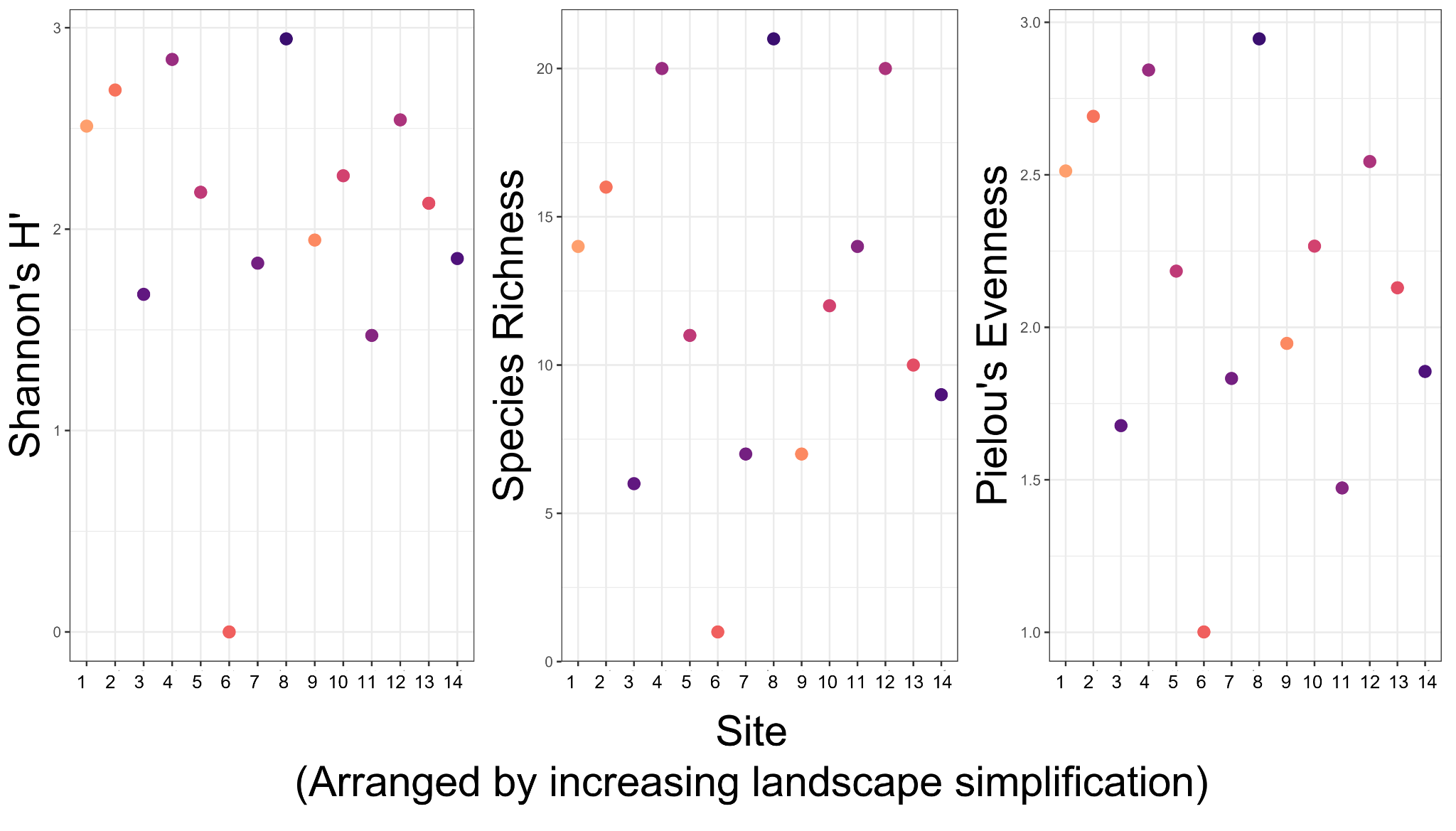


**Fig. S1** Species diversity (Shannon’s H’), richness, and evenness for bees and hoverflies collected from resident *B. vulgaris* plants adjacent to the focal population (to prevent damaging the plants when sweep netting pollinators). Each point represents a site arranged in order of increasing agricultural landscape simplification.

**Table S1**. Number of replicates of phytometer plants in each treatment combination for each site. Herbivore exclusion was implemented by placing mesh bags over the foliar rosette of 7 plants at each site. Once a plant bloomed, the bag was opened to allow the flowers to develop normally and re-tied beneath the inflorescence to continue excluding herbivores from the rosette. Pollinator exclusion was achieved by placing fine mesh bags over one inflorescence on each plant when possible based on the timing of visits (i.e. plants that already had most flowers open upon the first visit after bolting did not receive a pollinator exclusion bag) and availability of inflorescences (i.e. plants with only one main inflorescence did not receive a pollinator exclusion bag). Pollinator exclusion did not result in exclusion of pollinators from an entire plant but rather a portion to measure the contribution of pollinators to seed set within a plant.

| **Site** | **Herbivore Exclusion** | | **No Herbivore Exclusion** | |
| --- | --- | --- | --- | --- |
|  | **Pollinator Exclusion** | **No Pollinator Exclusion** | **Pollinator Exclusion** | **No Pollinator Exclusion** |
| Site 1 | 6 | 1 | 5 | 3 |
| Site 2 | 5 | 2 | 5 | 3 |
| Site 3 | 4 | 3 | 4 | 4 |
| Site 4 | 1 | 6 | 6 | 2 |
| Site 5 | 2 | 4 | 4 | 4 |
| Site 6 | 4 | 3 | 5 | 3 |
| Site 7 | 4 | 3 | 8 | 0 |
| Site 8 | 5 | 2 | 7 | 1 |
| Site 9 | 4 | 3 | 5 | 3 |
| Site 10 | 5 | 2 | 7 | 1 |
| Site 11 | 2 | 5 | 3 | 5 |
| Site 12 | 5 | 2 | 4 | 4 |

**Table S2**. Results of simple linear models and linear mixed-effects models with site included as a random effect for phytometer plants open to herbivory. These represent individual linear regressions for each path included in the path analysis to evaluate how the inclusion of site as a random effect influences the model output, because random effects cannot be included in the SEM framework in ‘lavaan’. Landscape refers to PC1 values from a principal component analysis summarizing pasture, natural, and agricultural land cover at three scales (500, 1000, and 1500m) for each site. Higher values indicate a greater proportion of agriculture in the surrounding landscape and lower values indicate a greater proportion of open (non-forested) natural area and pasture. Statistically significant predictors are indicated in bold (P < 0.05) and marginally significant predictors are indicated in italics (P < 0.1).

|  |  | **Linear Model (lm)** | | | **Linear Mixed-Effects Model (lmer)** | | | |
| --- | --- | --- | --- | --- | --- | --- | --- | --- |
| **Response** | **Predictors** | **Estimate (SE)** | **t** | **P** | **Estimate (SE)** | **df** | **t** | **P** |
| Total Seed Set | Damage | -0.02(0.11) | -0.15 | 0.881 | 0.01(0.12) | 56.00 | 0.107 | 0.92 |
|  | **Pollinator Contribution** | **2.75(0.42)** | **6.63** | **1.24e-8** | **2.41(0.48)** | **45.44** | **5.07** | **7.07e-6** |
|  | Landscape | -0.05(0.05) | -0.95 | 0.35 | -0.05(0.07) | 10.62 | -0.76 | 0.47 |
|  | **Flower Number** | **1.49(0.10)** | **15.19** | **<2e-16** | **1.45(0.10)** | **57.69** | **14.88** | **<2e-16** |
| Pollinator Contribution | Landscape | -0.01(0.01) | -1.09 | 0.28 | -0.02(0.02) | 10.56 | -0.85 | 0.42 |
|  | Flower Number | 0.03(0.03) | 1.15 | 0.26 | 0.02(0.03) | 52.03 | 0.92 | 0.36 |
|  | **Flower Size** | **0.08(0.03)** | **3.19** | **0.002** | **0.05(0.02)** | **49.77** | **2.47** | **0.02** |
| Flower Number | Damage | 0.18(0.11) | 1.57 | 0.12 | 0.19(0.12) | 70.38 | 1.56 | 0.12 |
|  | **Foliar Biomass** | **0.08(0.03)** | **2.65** | **0.01** | **0.07(0.03)** | **83.86** | **2.20** | **0.03** |
| Flower Size | **Damage** | **0.28(0.12)** | **2.41** | **0.02** | *0.21(0.13)* | *71.23* | *1.67* | *0.1* |

**Table S3**. Results of simple linear models and linear mixed-effects models with site included as a random effect for herbivore excluded phytometer plants. These represent individual linear regressions for each path included in the path analysis to evaluate how the inclusion of site as a random effect influences the model output, because random effects cannot be included in the SEM framework in ‘lavaan’. Landscape refers to PC1 values from a principal component analysis summarizing pasture, natural, and agricultural land cover at three scales (500, 1000, and 1500m) for each site. Higher values indicate a greater proportion of agriculture in the surrounding landscape and lower values indicate a greater proportion of open (non-forested) natural area and pasture. Statistically significant predictors are indicated in bold (P < 0.05) and marginally significant predictors are indicated in italics (P < 0.1).

|  |  | **Linear Model (lm)** | | | **Linear Mixed-Effects Model (lmer)** | | | |
| --- | --- | --- | --- | --- | --- | --- | --- | --- |
| **Response** | **Predictors** | **Estimate (SE)** | **t** | **P** | **Estimate (SE)** | **df** | **t** | **P** |
| Total Seed Set | Damage | -0.04(0.12) | -0.30 | 0.76 | -0.04(0.12) | 42.00 | -0.30 | 0.76 |
|  | **Pollinator Contribution** | **2.36(0.44)** | **5.31** | **3.87e-6** | **2.36(0.44)** | **42.00** | **5.31** | **3.87e-6** |
|  | Landscape | -0.06(0.05) | -1.31 | 0.20 | -0.06(0.05) | 42.00 | -1.31 | 0.20 |
|  | **Flower Number** | **1.44(0.10)** | **14.12** | **<2e-16** | **1.44(0.10)** | **42.00** | **14.12** | **<2e-16** |
| Pollinator Contribution | Landscape | -0.001(0.02) | -0.08 | 0.93 | -0.02(0.03) | 8.82 | 0.67 | 0.52 |
|  | **Flower Number** | **0.10(0.03)** | **3.18** | **0.003** | **0.07(0.03)** | **31.89** | **2.59** | **0.01** |
|  | **Flower Size** | **0.09(0.03)** | **2.53** | **0.016** | **0.07(0.03)** | **31.60** | **2.23** | **0.03** |
| Flower Number | Damage | -0.02(0.12) | -0.19 | 0.85 | 0.16(0.13) | 71.34 | 1.23 | 0.22 |
|  | **Foliar Biomass** | 0.04(0.03) | 1.45 | 0.15 | **0.06(0.02)** | **72.88** | **2.25** | **0.03** |
| Flower Size | Damage | 0.13(0.14) | 0.88 | 0.38 | 0.06(0.15) | 51.22 | 0.40 | 0.69 |

**Table S4**. Results of simple linear models and linear mixed-effects models with site included as a random effect for resident plants. These represent individual linear regressions for each path included in the path analysis to evaluate how the inclusion of site as a random effect influences the model output, because random effects cannot be included in the SEM framework in ‘lavaan’. Landscape refers to PC1 values from a principal component analysis summarizing pasture, natural, and agricultural land cover at three scales (500, 1000, and 1500m) for each site. Higher values indicate a greater proportion of agriculture in the surrounding landscape and lower values indicate a greater proportion of open (non-forested) natural area and pasture. Statistically significant predictors are indicated in bold (P < 0.05) and marginally significant predictors are indicated in italics (P < 0.1).

|  |  | **Linear Model (lm)** | | | **Linear Mixed-Effects Model (lmer)** | | | |
| --- | --- | --- | --- | --- | --- | --- | --- | --- |
| **Response** | **Predictors** | **Estimate (SE)** | **t** | **P** | **Estimate (SE)** | **df** | **t** | **P** |
| Total Seed Set | Damage | 0.34(0.35) | 0.99 | 0.33 | 0.34(0.35) | 71.00 | 0.99 | 0.33 |
|  | **Pollinator Contribution** | **8.35(1.15)** | **7.29** | **3.46e-10** | **8.35(1.15)** | **71.00** | **7.29** | **3.46e-10** |
|  | Landscape | 0.13(0.15) | 0.89 | 0.38 | 0.13(0.15) | 71.00 | 0.89 | 0.38 |
|  | **Flower Number** | **6.35(0.28)** | **22.57** | **<2e-16** | **6.35(0.28)** | **71.00** | **22.57** | **<2e-16** |
| Pollinator Contribution | Landscape | -0.01(0.02) | -0.64 | 0.52 | -0.01(0.03) | 9.00 | -0.27 | 0.79 |
|  | **Flower Number** | 0.05(0.03) | 1.54 | 0.13 | **0.08(0.03)** | **40.53** | **2.44** | **0.02** |
|  | Flower Size | 0.02(0.04) | 0.45 | 0.66 | 0.04(0.04) | 37.91 | 0.98 | 0.33 |
| Flower Number | Damage | 0.02(0.07) | 0.27 | 0.78 | -0.05(0.08) | 126.78 | -0.65 | 0.52 |
| Flower Size | Damage | 0.07(0.08) | 0.82 | 0.42 | 0.09(0.09) | 110.65 | 0.96 | 0.34 |

**Table S5.** Insects collected on flowers of *Barbarea vulgaris* by sweep netting resident plants adjacent to focal populations at 14 sites along a gradient of increasing agricultural landscape simplification. Each insect was identified to the highest classification possible, given available expertise. Because insects were collected via sweep netting, this list represents insects present on the flowers, but does not inform which species were actively pollinating when collected.

| **Family** | **Genus** | **Species** | **Number Collected** | **Identification Material** |
| --- | --- | --- | --- | --- |
| **Order: Hymenoptera** | | | | |
| Andrenidae | *Andrena* | *arabis* | 11 | Ribble, D. W. A Revision of the Bees of the Genus Andrena of the Western Hemisphere Subgenus  Scaphandrena. Trans. Am. Entomol. Soc. 100, 101-189 (1974) |
|  |  | *carlini* | 11 | Bouseman, J. K. & LaBerge, W. E. A revision of the bees of the genus Andrena of the Western Hemisphere. Part IX. Subgenus Melandrena. Trans. Am. Entomol. Soc. 104, 275– 389 (1979). |
|  |  | *ceanothi* | 3 | LaBerge, W. E. A Revision of the Bees of the Genus Andrena of the Western Hemisphere. Part VI.  Subgenus Trachandrena. Trans. Am. Entomol. Soc. 99, 235-371 (1973) |
|  |  | *crataegi* | 10 | LaBerge, W. E. A Revision of the Bees of the Genus Andrena of the Western Hemisphere. Part II.  Plastandrena, Aporandrena, Charitandrena. Trans. Am. Entomol. Soc. 95, 1-47 (1969) |
|  |  | *cressonii* | 3 | LaBerge, W. E. A revision of the bees of the genus Andrena of the Western Hemisphere. Part XI. Minor subgenera and subgeneric key. Trans. Am. Entomol. Soc. 111, 440–567 |
|  |  | *erythronii* | 1 | LaBerge, W. E. A Revision of the Bees of the Genus Andrena of the Western Hemisphere. Part XII.  Subgenera Leucandrena, Ptilandrena, Scoliandrena and Melandrena. Trans. Am. Entomol. Soc. 112, 191-248 (1986) |
|  |  | *hippotes* | 15 | LaBerge, W. E. A Revision of the Bees of the Genus Andrena of the Western Hemisphere. Part VI.  Subgenus Trachandrena. Trans. Am. Entomol. Soc. 99, 235-371 (1973) |
|  |  | *imitatrix* | 1 | LaBerge, W. E. A revision of the bees of the genus Andrena of the Western Hemisphere. Part IV. Scrapteropsis, Xiphandrena and Raphandrena. Trans. Am. Entomol. Soc. 97, 441–520 (1971). |
|  |  | *medium sp.* | 2 (male) |  |
|  |  | *nasonii* | 19 | LaBerge, W.E. A Revision of the Bees of the Genus Andrena of the Western Hemisphere. Part XIII. Subgenera Simandrena and Taeniandrena. Trans. Am. Entomol. Soc. 115, 1-56 (1989) |
|  |  | *nuda* | 1 | LaBerge, W. E. A Revision of the Bees of the Genus Andrena of the Western Hemisphere. Part VI.  Subgenus Trachandrena. Trans. Am. Entomol. Soc. 99, 235-371 (1973) |
|  |  | *perplexa* | 1 | Bouseman, J. K. & LaBerge, W. E. A revision of the bees of the genus Andrena of the Western Hemisphere. Part IX. Subgenus Melandrena. Trans. Am. Entomol. Soc. 104, 275– 389 (1979). |
|  |  | *regularis* | 10 | Bouseman, J. K. & LaBerge, W. E. A revision of the bees of the genus Andrena of the Western Hemisphere. Part IX. Subgenus Melandrena. Trans. Am. Entomol. Soc. 104, 275– 389 (1979). |
|  |  | *rufosignata* | 4 | LaBerge, W. E. A Revision of the Bees of the Genus Andrena of the Western Hemisphere. Part X.  Subgenus Andrena. Trans. Am. Entomol. Soc. 106, 395-525 (1980) |
|  |  | *rugosa* | 4 | LaBerge, W. E. A Revision of the Bees of the Genus Andrena of the Western Hemisphere. Part VI.  Subgenus Trachandrena. Trans. Am. Entomol. Soc. 99, 235-371 (1973) |
|  |  | *sigmundi* | 1 | LaBerge, W. E. A Revision of the Bees of the Genus Andrena of the Western Hemisphere. Part VI.  Subgenus Trachandrena. Trans. Am. Entomol. Soc. 99, 235-371 (1973) |
|  |  | *small sp.* | 1 (male) |  |
|  |  | *tridens* | 1 | LaBerge, W. E. A Revision of the Bees of the Genus Andrena of the Western Hemisphere. Part X.  Subgenus Andrena. Trans. Am. Entomol. Soc. 106, 395-525 (1980) |
|  |  | *vicina* | 1 | Bouseman, J. K. & LaBerge, W. E. A revision of the bees of the genus Andrena of the Western Hemisphere. Part IX. Subgenus Melandrena. Trans. Am. Entomol. Soc. 104, 275– 389 (1979). |
|  |  | *wheeleri* | 2 | LaBerge, W.E. A Revision of the Bees of the Genus Andrena of the Western Hemisphere. Part XIII. Subgenera Simandrena and Taeniandrena. Trans. Am. Entomol. Soc. 115, 1-56 (1989) |
| Apidae | *Apis* | *mellifera* | 29 |  |
|  | *Bombus* | *vagans* | 1 | Williams, P. H., Thorp, R. W., Richardson, L. L. & Colla, S. R. Bumble bees of North America: An identification guide. (Princeton University Press, 2014); Laverty, T. M. & Harder, L. D. The bumble 25 bees of eastern Canada. Can. Entomol. 120, 965–967 (1988). |
|  | *Ceratina* | *calcarata* | 5 | Rehan, S. M. & Sheffield, C. S. Morphological and molecular delineation of a new species in the Ceratina dupla species-group (Hymenoptera: Apidae: Xylocopinae) of eastern North America. Zootaxa 2873, 35– 50 (2011). |
|  |  | *dupla* | 2 | Rehan, S. M. & Sheffield, C. S. Morphological and molecular delineation of a new species in the Ceratina dupla species-group (Hymenoptera: Apidae: Xylocopinae) of eastern North America. Zootaxa 2873, 35– 50 (2011). |
|  |  | *mikmaqi* | 1 | Rehan, S. M. & Sheffield, C. S. Morphological and molecular delineation of a new species in the Ceratina dupla species-group (Hymenoptera: Apidae: Xylocopinae) of eastern North America. Zootaxa 2873, 35– 50 (2011). |
|  | *Nomada* | *sp.* | 6 (male) |  |
| Braconidae | *Peristenus* | *sp.* | 1 | Sharkey, M.J. Key to New World Subfamilies of the Family Braconidae. in *Manual of the New World Genera of the Family Braconidae* (eds. Wharton, R.A., Marsh, P.M., and Sharkey, M.J.) 39-64 (Allen Press, 1997). |
|  |  |  | 2 | Sharkey, M.J. Key to New World Subfamilies of the Family Braconidae. in *Manual of the New World Genera of the Family Braconidae* (eds. Wharton, R.A., Marsh, P.M., and Sharkey, M.J.) 39-64 (Allen Press, 1997). |
| Colletidae | *Colletes* | *inaequalis* | 1 | Mitchell, T. B. Bees of the Eastern United States: volume I. N. C. Agric. Exp. Stn. Tech. Bull. 141, 1–538 (1960); Stephen, W. P. A revision of the bee genus Colletes in America North of Mexico. Univ. Kansas Sci. Bull. 36, 149–527 (1954). |
| Eulophidae | *Closterocerus* | *sp.* | 1 | Schauff, M.E., LaSalle, J., Coote, L.D. Chapter 10. Eulophidae. in *Annotated Keys to the Genera of Nearctic Chalcidoidea (Hymenoptera)* (eds. Gibson, G.A.P., Huber, J.T., Woolley, J.B.) 327-429 (National Research Council of Canada, 1997). |
|  | *Tetrastichus* | *sp.* | 1 | Schauff, M.E., LaSalle, J., Coote, L.D. Chapter 10. Eulophidae. in *Annotated Keys to the Genera of Nearctic Chalcidoidea (Hymenoptera)* (eds. Gibson, G.A.P., Huber, J.T., Woolley, J.B.) 327-429 (National Research Council of Canada, 1997). |
| Formicidae |  |  | 21 |  |
| Halictidae | *Augochlora* | *pura* | 1 | Mitchell, T. B. Bees of the Eastern United States: volume I. N. C. Agric. Exp. Stn. Tech. Bull. 141, 1–538 (1960). |
|  | *Augochlorella* | *aurata* | 14 | Coelho, B. W. T. A review of the bee genus Augochlorella (Hymenoptera: Halictidae: Augochlorini). Syst. Entomol. 29, 282–323 (2004). |
|  | *Augochloropsis* | *metallica* | 1 | Mitchell, T. B. Bees of the Eastern United States: volume I. N. C. Agric. Exp. Stn. Tech. Bull. 141, 1–538 (1960). |
|  | *Halictus* | *confusus* | 5 | Mitchell, T. B. Bees of the Eastern United States: volume I. N. C. Agric. Exp. Stn. Tech. Bull. 141, 1–538 (1960). |
|  |  | *ligatus* | 2 | Mitchell, T. B. Bees of the Eastern United States: volume I. N. C. Agric. Exp. Stn. Tech. Bull. 141, 1–538 (1960). |
|  |  | *rubicundus* | 7 | Mitchell, T. B. Bees of the Eastern United States: volume I. N. C. Agric. Exp. Stn. Tech. Bull. 141, 1–538 (1960). |
|  | *Lasioglossum* | *cattellae* | 2 | Gibbs, J. Revision of the metallic Lasioglossum (Dialictus) of eastern North America (Hymenoptera: Halictidae: Halictini). Zootaxa 1–216 (2011). |
|  |  | *coreopsis* | 1 | Gibbs, J. Revision of the metallic Lasioglossum (Dialictus) of eastern North America (Hymenoptera: Halictidae: Halictini). Zootaxa 1–216 (2011). |
|  |  | *coriaceum* | 1 | McGinley, R. J. Studies of Halictinae (Apoidea: Halictidae), I: Revision of New World Lasioglossum Curtis. Smithson. Contrib. to Zool. 429, 1–294 (1986). |
|  |  | *cressonii* | 1 | Gibbs, J. Revision of the metallic Lasioglossum (Dialictus) of eastern North America (Hymenoptera: Halictidae: Halictini). Zootaxa 1–216 (2011). |
|  |  | *ephilatum* | 3 | Gibbs, J. Revision of the metallic Lasioglossum (Dialictus) of eastern North America (Hymenoptera: Halictidae: Halictini). Zootaxa 1–216 (2011). |
|  |  | *foxii* | 9 | Gibbs, J., Packer, L., Dumesh, S. & Danforth, B. N. Revision and reclassification of Lasioglossum (Evylaeus), L. (Hemihalictus) and L. (Sphecodogastra) in eastern North America (Hymenoptera: Apoidea: Halictidae). Zootaxa 3672, 1– 117 (2013). |
|  |  | *georgeickworti* | 3 | Gibbs, J. Revision of the metallic Lasioglossum (Dialictus) of eastern North America (Hymenoptera: Halictidae: Halictini). Zootaxa 1–216 (2011). |
|  |  | *imitatum* | 5 | Gibbs, J. Revision of the metallic Lasioglossum (Dialictus) of eastern North America (Hymenoptera: Halictidae: Halictini). Zootaxa 1–216 (2011). |
|  |  | *laevissimum* | 2 | Gibbs, J. Revision of the metallic Lasioglossum (Dialictus) of eastern North America (Hymenoptera: Halictidae: Halictini). Zootaxa 1–216 (2011). |
|  |  | *lineatulum* | 2 | Gibbs, J. Revision of the metallic Lasioglossum (Dialictus) of eastern North America (Hymenoptera: Halictidae: Halictini). Zootaxa 1–216 (2011). |
|  |  | *nymphaearum* | 2 | Gibbs, J. Revision of the metallic Lasioglossum (Dialictus) of eastern North America (Hymenoptera: Halictidae: Halictini). Zootaxa 1–216 (2011). |
|  |  | *obscurum* | 10 | Gibbs, J. Revision of the metallic Lasioglossum (Dialictus) of eastern North America (Hymenoptera: Halictidae: Halictini). Zootaxa 1–216 (2011). |
|  |  | *paradmirandum* | 7 | Gibbs, J. Revision of the metallic Lasioglossum (Dialictus) of eastern North America (Hymenoptera: Halictidae: Halictini). Zootaxa 1–216 (2011). |
|  |  | *pectorale* | 1 | Gibbs, J., Packer, L., Dumesh, S. & Danforth, B. N. Revision and reclassification of Lasioglossum (Evylaeus), L. (Hemihalictus) and L. (Sphecodogastra) in eastern North America (Hymenoptera: Apoidea: Halictidae). Zootaxa 3672, 1– 117 (2013). |
|  |  | *perpunctatum* | 10 | Gibbs, J. Revision of the metallic Lasioglossum (Dialictus) of eastern North America (Hymenoptera: Halictidae: Halictini). Zootaxa 1–216 (2011). |
|  |  | *pilosum* | 2 | Gibbs, J. Revision of the metallic Lasioglossum (Dialictus) of eastern North America (Hymenoptera: Halictidae: Halictini). Zootaxa 1–216 (2011). |
|  |  | *planatum* | 3 | Gibbs, J. Revision of the metallic Lasioglossum (Dialictus) of eastern North America (Hymenoptera: Halictidae: Halictini). Zootaxa 1–216 (2011). |
|  |  | *quebecense* | 1 | Gibbs, J., Packer, L., Dumesh, S. & Danforth, B. N. Revision and reclassification of Lasioglossum (Evylaeus), L. (Hemihalictus) and L. (Sphecodogastra) in eastern North America (Hymenoptera: Apoidea: Halictidae). Zootaxa 3672, 1– 117 (2013). |
|  |  | *subviridatum* | 1 | Gibbs, J. Revision of the metallic Lasioglossum (Dialictus) of eastern North America (Hymenoptera: Halictidae: Halictini). Zootaxa 1–216 (2011). |
|  |  | *tegulare* | 2 | Gibbs, J. Revision of the metallic Lasioglossum (Dialictus) of eastern North America (Hymenoptera: Halictidae: Halictini). Zootaxa 1–216 (2011). |
|  |  | *versans* | 1 | Gibbs, J. Revision of the metallic Lasioglossum (Dialictus) of eastern North America (Hymenoptera: Halictidae: Halictini). Zootaxa 1–216 (2011). |
|  |  | *versatum* | 11 | Gibbs, J. Revision of the metallic Lasioglossum (Dialictus) of eastern North America (Hymenoptera: Halictidae: Halictini). Zootaxa 1–216 (2011). |
|  |  | *hitchensi/*  *weemsi* | 6 | Gibbs, J. Revision of the metallic Lasioglossum (Dialictus) of eastern North America (Hymenoptera: Halictidae: Halictini). Zootaxa 1–216 (2011). |
|  |  | *zephyrum* | 2 | Gibbs, J. Revision of the metallic Lasioglossum (Dialictus) of eastern North America (Hymenoptera: Halictidae: Halictini). Zootaxa 1–216 (2011). |
|  |  | *zonulum* | 2 | McGinley, R. J. Studies of Halictinae (Apoidea: Halictidae), I: Revision of New World Lasioglossum Curtis. Smithson. Contrib. to Zool. 429, 1–294 (1986). |
| Ichneumonidae |  |  | 7 |  |
| Pteromalidae | *Colotrechnus* | *sp.* | 5 | Bouček, Z., Heydon, S.L. Chapter 17. Pteromalidae. in *Annotated Keys to the Genera of Nearctic Chalcidoidea (Hymenoptera)* (eds. Gibson, G.A.P., Huber, J.T., Woolley, J.B.) 541-692 (National Research Council of Canada, 1997). |
|  | *Macromesus* | *sp.* | 6 | Bouček, Z., Heydon, S.L. Chapter 17. Pteromalidae. in *Annotated Keys to the Genera of Nearctic Chalcidoidea (Hymenoptera)* (eds. Gibson, G.A.P., Huber, J.T., Woolley, J.B.) 541-692 (National Research Council of Canada, 1997). |
|  |  |  | 2 |  |
| Tetracampidae | *Platynocheilus* | *sp.* | 3 | Bouček, Z. Chapter 20. Tetracampidae. in *Annotated Keys to the Genera of Nearctic Chalcidoidea (Hymenoptera)* (eds. Gibson, G.A.P., Huber, J.T., Woolley, J.B.) 705-708 (National Research Council of Canada, 1997). |
| Vespidae | *Vespula* | *flavopilosa* | 1 | Buck, M., Marshall, S.A., Cheung, D.K.B. Identification Atlas of the Vespidae (Hymenoptera, Aculeata) of the Northeastern Nearctic Region. *Canadian Journal of Arthropod Identification*. 05 (2008). |
| **Order: Diptera** | | | | |
| Agromyzidae | *Cerodontha* | *dorsalis* | 1 | Lonsdale, O. Manual of North American Agromyzidae (Diptera, Schizophora), with revision of the fauna of the “Delmarva” states. *Zookeys*. 1051, 1-481 (2021). |
| Anthomyiidae | *Delia* | *platura* | 48 | Savage, J., Fortier, A., Fournier, F., Bellavance, V. Identification of *Delia* pest species (Diptera: Anthomyiidae) in cultivated crucifers and other vegetable crops in Canada. *Canadian Journal of Arthropod Identification*. 29 (2016).. |
|  |  |  | 3 | Huckett, H.C. Chapter One Hundred Four - Anthomyiidae. in *Manual of Nearctic Diptera* (coors. McAlpine, J.F., Peterson, B.V., Shewell, G.E., Teskey, H.J., Vockeroth, J.R., Wood, D.M.) (ed. McAlpine, J.F.) vol. 2 1099-1114 (Biosystematics Research Institute, Ottawa, 1981). |
| Bibionidae | *Bibio* | *sp.* | 4 | Hardy, D.E. Chapter Thirteen - Bibionidae. in *Manual of Nearctic Diptera* (coors. McAlpine, J.F., Peterson, B.V., Shewell, G.E., Teskey, H.J., Vockeroth, J.R., and Wood, D.M.) vol. 1 217-222 (Biosystematics Research Institute, Ottawa, 1981). |
| Calliphoridae | *Lucilia* | *sp.* | 1 | Shewell, G.E. Chapter One Hundred Six - Calliphoridae. in *Manual of Nearctic Diptera* (coors. McAlpine, J.F., Peterson, B.V., Shewell, G.E., Teskey, H.J., Vockeroth, J.R., Wood, D.M.) (ed. McAlpine, J.F.) vol. 2 1133-1145 (Biosystematics Research Institute, Ottawa, 1981). |
| Chloropidae | *Apallates* | *sp.* | 2 | Sabrosky, C.W. Chapter Ninety-Nine - Chloropidae. in *Manual of Nearctic Diptera* (coors. McAlpine, J.F., Peterson, B.V., Shewell, G.E., Teskey, H.J., Vockeroth, J.R., Wood, D.M.) (ed. McAlpine, J.F.) vol. 2 1049-1067 (Biosystematics Research Institute, Ottawa, 1981). |
|  | *Chlorops* | *sp.* | 1 | Sabrosky, C.W. Chapter Ninety-Nine - Chloropidae. in *Manual of Nearctic Diptera* (coors. McAlpine, J.F., Peterson, B.V., Shewell, G.E., Teskey, H.J., Vockeroth, J.R., Wood, D.M.) (ed. McAlpine, J.F.) vol. 2 1049-1067 (Biosystematics Research Institute, Ottawa, 1981). |
|  | *Dicraeus* | *sp.* | 2 | Sabrosky, C.W. Chapter Ninety-Nine - Chloropidae. in *Manual of Nearctic Diptera* (coors. McAlpine, J.F., Peterson, B.V., Shewell, G.E., Teskey, H.J., Vockeroth, J.R., Wood, D.M.) (ed. McAlpine, J.F.) vol. 2 1049-1067 (Biosystematics Research Institute, Ottawa, 1981). |
|  | *Elachiptera* | *sp.* | 4 | Sabrosky, C.W. Chapter Ninety-Nine - Chloropidae. in *Manual of Nearctic Diptera* (coors. McAlpine, J.F., Peterson, B.V., Shewell, G.E., Teskey, H.J., Vockeroth, J.R., Wood, D.M.) (ed. McAlpine, J.F.) vol. 2 1049-1067 (Biosystematics Research Institute, Ottawa, 1981). |
|  | *Oscinella* | *sp.* | 1 | Sabrosky, C.W. Chapter Ninety-Nine - Chloropidae. in *Manual of Nearctic Diptera* (coors. McAlpine, J.F., Peterson, B.V., Shewell, G.E., Teskey, H.J., Vockeroth, J.R., Wood, D.M.) (ed. McAlpine, J.F.) vol. 2 1049-1067 (Biosystematics Research Institute, Ottawa, 1981). |
|  | *Psilacrum* | *sp.* | 1 | Sabrosky, C.W. Chapter Ninety-Nine - Chloropidae. in *Manual of Nearctic Diptera* (coors. McAlpine, J.F., Peterson, B.V., Shewell, G.E., Teskey, H.J., Vockeroth, J.R., Wood, D.M.) (ed. McAlpine, J.F.) vol. 2 1049-1067 (Biosystematics Research Institute, Ottawa, 1981). |
|  | *Rhopalopterum* | *sp.* | 3 | Sabrosky, C.W. Chapter Ninety-Nine - Chloropidae. in *Manual of Nearctic Diptera* (coors. McAlpine, J.F., Peterson, B.V., Shewell, G.E., Teskey, H.J., Vockeroth, J.R., Wood, D.M.) (ed. McAlpine, J.F.) vol. 2 1049-1067 (Biosystematics Research Institute, Ottawa, 1981). |
|  | *Thaumatomyia* | *sp.* | 5 | Sabrosky, C.W. Chapter Ninety-Nine - Chloropidae. in *Manual of Nearctic Diptera* (coors. McAlpine, J.F., Peterson, B.V., Shewell, G.E., Teskey, H.J., Vockeroth, J.R., Wood, D.M.) (ed. McAlpine, J.F.) vol. 2 1049-1067 (Biosystematics Research Institute, Ottawa, 1981). |
|  |  |  | 1 |  |
| Conopidae | *Zodion* | *sp.* | 2 | Smith, K.G.V., Peterson, B.V. Chapter Fifty-Four - Conopidae. in *Manual of Nearctic Diptera* (coors. McAlpine, J.F., Peterson, B.V., Shewell, G.E., Teskey, H.J., Vockeroth, J.R., Wood, D.M.) (ed. McAlpine, J.F.) vol. 2 749-756 (Biosystematics Research Institute, Ottawa, 1981). |
| Culicidae | *Aedes* | *sp.* | 2 | Stone, A. Chapter Twenty-Five - Culicidae. in *Manual of Nearctic Diptera* (coors. McAlpine, J.F., Peterson, B.V., Shewell, G.E., Teskey, H.J., Vockeroth, J.R., Wood, D.M.) vol. 1 341-350 (Biosystematics Research Institute, Ottawa, 1981). |
|  | *Psorophora* | *sp.* | 3 | Stone, A. Chapter Twenty-Five - Culicidae. in *Manual of Nearctic Diptera* (coors. McAlpine, J.F., Peterson, B.V., Shewell, G.E., Teskey, H.J., Vockeroth, J.R., Wood, D.M.) vol. 1 341-350 (Biosystematics Research Institute, Ottawa, 1981). |
| Dolichopodidae | *Chrysotus* | *sp.* | 1 | Robinson, H., Vockeroth, J.R. Chapter Forty-Eight - Dolichopodidae. in *Manual of Nearctic Diptera* (coors. McAlpine, J.F., Peterson, B.V., Shewell, G.E., Teskey, H.J., Vockeroth, J.R., and Wood, D.M.) vol. 1 625-639 (Biosystematics Research Institute, Ottawa, 1981). |
| Empididae | *Hilara* | *sp.* | 1 | Steyskal, G.C., Knutson, L.V. Chapter Forty-Seven - Empididae. in *Manual of Nearctic Diptera* (coors. McAlpine, J.F., Peterson, B.V., Shewell, G.E., Teskey, H.J., Vockeroth, J.R., Wood, D.M.) vol. 1 607-624 (Biosystematics Research Institute, Ottawa, 1981). |
|  | *Rhamphomyia* | *sp.* | 1 | Steyskal, G.C., Knutson, L.V. Chapter Forty-Seven - Empididae. in *Manual of Nearctic Diptera* (coors. McAlpine, J.F., Peterson, B.V., Shewell, G.E., Teskey, H.J., Vockeroth, J.R., Wood, D.M.) vol. 1 607-624 (Biosystematics Research Institute, Ottawa, 1981). |
| Fanniidae | *Fannia* | *sp.* | 1 | Huckett, H.C, Vockeroth, J.R.. Chapter One Hundred Five - Muscidae. in *Manual of Nearctic Diptera* (coors. McAlpine, J.F., Peterson, B.V., Shewell, G.E., Teskey, H.J., Vockeroth, J.R., Wood, D.M.) (ed. McAlpine, J.F.) vol. 2 1115-1131 (Biosystematics Research Institute, Ottawa, 1981). |
| Hybotidae | *Platypalpus* | *sp.* | 2 | Steyskal, G.C., Knutson, L.V. Chapter Forty-Seven - Empididae. in *Manual of Nearctic Diptera* (coors. McAlpine, J.F., Peterson, B.V., Shewell, G.E., Teskey, H.J., Vockeroth, J.R., Wood, D.M.) vol. 1 607-624 (Biosystematics Research Institute, Ottawa, 1981). |
| Muscidae | *Coenosia* | *tigrina* | 4 | Huckett, H.C. ARevision of theNorth American Species Belongin to the Genus *Coenosia* Meigen and related genera (Diptera: Muscidae). Part I. The Subgenera *Neodexiopsis*, *Coenosia*, *Hoplogaster* and Related Genera *Allognota*, *Bithoracochaeta* and *Schoenomyza*. Transactions of the American Entomological Society. 60(2) 133-198 (1934). |
|  | *Eudasyphora* | *cyanicolor* | 1 | Cuny, R. Revision of the Genus *Eudasyphora* Townsend (Diptera: Muscidae), and Reflections on Its Evolution. *The Canadian Entomologist*. 112(4) (1980)). |
|  | *Myospila* | *meditabunda* | 1 | Huckett, H.C, Vockeroth, J.R.. Chapter One Hundred Five - Muscidae. in *Manual of Nearctic Diptera* (coors. McAlpine, J.F., Peterson, B.V., Shewell, G.E., Teskey, H.J., Vockeroth, J.R., Wood, D.M.) (ed. McAlpine, J.F.) vol. 2 1115-1131. |
| Rhinophoridae | *Stevenia* | *deceptoria* | 3 | O'Hara, J.E., Cerretti, P., Dahlem, G.A. First North American Record of the Palearctic Rhinophorid *Stevenia deceptoria* (Leow) (Diptera: Rhinophoridae). *Zootaxa*. 4058(2) 293-295 (2015). |
| Sarcophagidae | *Boettcheria* | *cimbicis* | 1 | Shewell, G.E. Chapter One Hundred Eight - Sarcophagidae. in *Manual of Nearctic Diptera* (coors. McAlpine, J.F., Peterson, B.V., Shewell, G.E., Teskey, H.J., Vockeroth, J.R., Wood, D.M.) (ed. McAlpine, J.F.) vol. 2 1159-1186 (Biosystematics Research Institute, Ottawa, 1981). |
|  | *Sarcophaga* | *sinuata* | 1 | Shewell, G.E. Chapter One Hundred Eight - Sarcophagidae. in *Manual of Nearctic Diptera* (coors. McAlpine, J.F., Peterson, B.V., Shewell, G.E., Teskey, H.J., Vockeroth, J.R., Wood, D.M.) (ed. McAlpine, J.F.) vol. 2 1159-1186 (Biosystematics Research Institute, Ottawa, 1981). |
|  |  | *sp.* | 8 | Shewell, G.E. Chapter One Hundred Eight - Sarcophagidae. in *Manual of Nearctic Diptera* (coors. McAlpine, J.F., Peterson, B.V., Shewell, G.E., Teskey, H.J., Vockeroth, J.R., Wood, D.M.) (ed. McAlpine, J.F.) vol. 2 1159-1186 (Biosystematics Research Institute, Ottawa, 1981). |
| Scathophagidae | *Scathophaga* | *stercoraria* | 10 | Vockeroth, J.R. Chapter One Hundred Three - Scathophagidae. in *Manual of Nearctic Diptera* (coors. McAlpine, J.F., Peterson, B.V., Shewell, G.E., Teskey, H.J., Vockeroth, J.R., Wood, D.M.) (ed. McAlpine, J.F.) vol. 2 1085-1097 (Biosystematics Research Institute, Ottawa, 1981). |
|  |  | *sp.* | 5 | Vockeroth, J.R. Chapter One Hundred Three - Scathophagidae. in *Manual of Nearctic Diptera* (coors. McAlpine, J.F., Peterson, B.V., Shewell, G.E., Teskey, H.J., Vockeroth, J.R., Wood, D.M.) (ed. McAlpine, J.F.) vol. 2 1085-1097 (Biosystematics Research Institute, Ottawa, 1981). |
| Sciaridae | *Sciara* | *sp.* | 1 | Steffan, W.A. Chapter Fifteen - Sciaridae. in *Manual of Nearctic Diptera* (coors. McAlpine, J.F., Peterson, B.V., Shewell, G.E., Teskey, H.J., Vockeroth, J.R., Wood, D.M.) vol. 1 247-255 (Biosystematics Research Institute, Ottawa, 1981). |
| Sepsidae | *Sepsis* | *punctum* | 3 | Pont, A.C. & Meier, R. The Sepsidae (Diptera) of Europe. *Fauna Entomologica Scandinavica.* 37 (2002). |
|  | *Themira* | *sp.* | 13 | Steyskal, G. Chapter Eighty Six - Sepsidae. in *Manual of Nearctic Diptera* (coors. McAlpine, J.F., Peterson, B.V., Shewell, G.E., Teskey, H.J., Vockeroth, J.R., Wood, D.M.) (ed. McAlpine, J.F.) vol. 2 945-950 (Biosystematics Research Institute, Ottawa, 1981). |
| Simuliidae | *Prosimulium* | *sp.* | 1 | Peterson, B.V. Chapter Twenty-Seven - Simuliidae. in *Manual of Nearctic Diptera* (coors. McAlpine, J.F., Peterson, B.V., Shewell, G.E., Teskey, H.J., Vockeroth, J.R., Wood, D.M.) vol. 1 355-391 (Biosystematics Research Institute, Ottawa, 1981). |
|  | *Simulium* | *sp.* | 6 | Peterson, B.V. Chapter Twenty-Seven - Simuliidae. in *Manual of Nearctic Diptera* (coors. McAlpine, J.F., Peterson, B.V., Shewell, G.E., Teskey, H.J., Vockeroth, J.R., Wood, D.M.) vol. 1 355-391 (Biosystematics Research Institute, Ottawa, 1981). |
| Stratiomyiidae | *Allognosta* | *fuscitarsis* | 34 | Woodley, N. A Revision of the Nearctic Berdinae (Diptera: Stratiomyiidae). Bulletin of the Museum of Comparative Zoology. 149(6) (1981). |
|  | *Microchrysa* | *polita* | 3 | James, M.T. Chapter Thirty-Nine - Stratiomyiidae. in *Manual of Nearctic Diptera* (coors. McAlpine, J.F., Peterson, B.V., Shewell, G.E., Teskey, H.J., Vockeroth, J.R., Wood, D.M.) vol. 1 497-523 (Biosystematics Research Institute, Ottawa, 1981). |
| Syrphidae | *Dasysyrphus* | *intrudens* | 1 | Locke, M.M., Skevington, J.H. Revision of Nearctic *Dasysyrphus* Enderlein (Diptera: Syrphidae).Zootaxa. 3660(1) 1-80 (2013). |
|  | *Eristalinus* | *aeneus* | 1 | Miranda, G.F.G., Young, A.D., Locke, M.M., Marshall, S.A., Skevington, J.H., Thompson, F.C. Key to the Genera of Nearctic Syrphidae. Canadian Journal of Arthropod Identification. 23 (2013). |
|  | *Eristalis* | *arbustorum* | 90 | Telford, H.S. *Eristalis* (Diptera: Syrphidae) From America North of Mexico. Annals of the Entomological Society of America. 63(5) 1201-1210 (1971). |
|  |  | *brousii* | 3 | Telford, H.S. *Eristalis* (Diptera: Syrphidae) From America North of Mexico. Annals of the Entomological Society of America. 63(5) 1201-1210 (1971). |
|  |  | *cryptarum* | 1 | Telford, H.S. *Eristalis* (Diptera: Syrphidae) From America North of Mexico. Annals of the Entomological Society of America. 63(5) 1201-1210 (1971). |
|  |  | *dimidiata* | 10 | Telford, H.S. *Eristalis* (Diptera: Syrphidae) From America North of Mexico. Annals of the Entomological Society of America. 63(5) 1201-1210 (1971). |
|  |  | *tenax* | 12 | Telford, H.S. *Eristalis* (Diptera: Syrphidae) From America North of Mexico. Annals of the Entomological Society of America. 63(5) 1201-1210 (1971). |
|  |  | *transversa* | 6 | Telford, H.S. *Eristalis* (Diptera: Syrphidae) From America North of Mexico. Annals of the Entomological Society of America. 63(5) 1201-1210 (1971). |
|  | *Eupeodes* | *americanus* | 1 | Vockeroth, J.R. The Flower Flies of the Subfamily Syrphinae of Canada, Alaska and Greenland. (Centre for Land and Biological Resources Research, Ottawa, Canada, 1992). |
|  | *Sphaerophoria* | *asymmetrica* | 1 | Vockeroth, J.R. The Flower Flies of the Subfamily Syrphinae of Canada, Alaska and Greenland. (Centre for Land and Biological Resources Research, Ottawa, Canada, 1992). |
|  |  | *philanthus* | 1 | Vockeroth, J.R. The Flower Flies of the Subfamily Syrphinae of Canada, Alaska and Greenland. (Centre for Land and Biological Resources Research, Ottawa, Canada, 1992). |
|  |  | *sp.* | 3 | Vockeroth, J.R. The Flower Flies of the Subfamily Syrphinae of Canada, Alaska and Greenland. (Centre for Land and Biological Resources Research, Ottawa, Canada, 1992). |
|  | *Syritta* | *pipiens* | 3 | Vockeroth, J.R. The Flower Flies of the Subfamily Syrphinae of Canada, Alaska and Greenland. (Centre for Land and Biological Resources Research, Ottawa, Canada, 1992). |
|  | *Syrphus* | *sp.* | 1 | Vockeroth, J.R. The Flower Flies of the Subfamily Syrphinae of Canada, Alaska and Greenland. (Centre for Land and Biological Resources Research, Ottawa, Canada, 1992). |
|  | *Toxomerus* | *geminatus* | 9 | Vockeroth, J.R. The Flower Flies of the Subfamily Syrphinae of Canada, Alaska and Greenland. (Centre for Land and Biological Resources Research, Ottawa, Canada, 1992). |
|  |  | *marginatus* | 15 | Vockeroth, J.R. The Flower Flies of the Subfamily Syrphinae of Canada, Alaska and Greenland. (Centre for Land and Biological Resources Research, Ottawa, Canada, 1992). |
| Tabanidae | *Chrysops* | *sp.* | 1 | Pechuman, L.L., Teskey, H.J. Chapter Thirty-One - Tabanidae. in *Manual of Nearctic Diptera* (coors. McAlpine, J.F., Peterson, B.V., Shewell, G.E., Teskey, H.J., Vockeroth, J.R., Wood, D.M.) vol. 1 463-478 (Biosystematics Research Institute, Ottawa, 1981). |
| Tachinidae | *Strongygaster* | *sp.* | 1 | Wood, D.M. Chapter One Hundred Ten - Tachinidae. in *Manual of Nearctic Diptera* (coors. McAlpine, J.F., Peterson, B.V., Shewell, G.E., Teskey, H.J., Vockeroth, J.R., Wood, D.M.) (ed. McAlpine, J.F.) vol. 2 1193-1269 (Biosystematics Research Institute, Ottawa, 1981). |
|  | *Xanthophyto* | *sp.* | 1 | Wood, D.M. Chapter One Hundred Ten - Tachinidae. in *Manual of Nearctic Diptera* (coors. McAlpine, J.F., Peterson, B.V., Shewell, G.E., Teskey, H.J., Vockeroth, J.R., Wood, D.M.) (ed. McAlpine, J.F.) vol. 2 1193-1269 (Biosystematics Research Institute, Ottawa, 1981). |
|  |  |  | 1 |  |
| Tipulidae | *Nephrotoma* | *sp.* | 1 | Alexander, C.P., Byers, G.W. Chapter Seven - Tipulidae. in *Manual of Nearctic Diptera* (coors. McAlpine, J.F., Peterson, B.V., Shewell, G.E., Teskey, H.J., Vockeroth, J.R., Wood, D.M.) vol. 1 153-190 (Biosystematics Research Institute, Ottawa, 1981). |
| Ulidiidae | *Chaetopsis* | *fulvifrons* | 5 | Johnson, C.W. Insects of Florida. American Mueum of Natural History. 32(3) 37-90 (1913). |
| **Order: Coleoptera** | | | | |
| Cantharidae | *Cantharis* | *livida* | 1 | Pelletier, G., Hébert, C. The Cantharidae of Eastern Canada and Northeastern United States. *Canadian Journal of Arthropod Identification*. 25 (2014). |
| Chrysomelidae | *Microrhopala* | *vittata* | 2 | Clark, S.M. A Revision of the Genus *Microrhopala* (Coleoptera: Chrysomelidae) in America North of Mexico. *The Great Basin Naturalist*. 43(4), 597-618 (1983). |
|  | *Neogalerucella* | *calmariensis* | 2 | Manguin, S., White, R., Blossey, B., Hight, S.D. Genetics, Taxonomy, and Ecology of Certain Species of *Galerucella* (Coleoptera: Chrysomelidae). Annals of the Entomological Society of America. 86(4) 397-410 (1993). |
|  | *Phyllotreta* | *striolata* | 2 | Smith, E.H. Revision of the Genus *Phyllotreta* Chevrolat of America North of Mexico. Field Museum of Natural History. (1985). |
|  | *Psylliodes* |  | 1 | Gilbert, A.J., Riley, E.G., Clark, S.M., Flowers, R.W. 124. Chrysomelidae Latreille 1802. in *American Beetles Volume 2 Polyphaga: Scarabaeoidea through Curculionoidea* (eds. Arnett, R.H., Thomas, M.C., Skelley P.E., Frank, J.H.) 617-691 (CRC Press, 2002). |
| Coccinellidae | *Coleomegilla* | *maculata* | 12 | Alyokhin, A., Donahue, C., Majka, C., Chandler, D.S., Hanley, G., Molengraaf, T., Beckendorf, E., Hesler, L. Ladybugs of Maine. Maine Agricultural and Forest Experiment Station. (2011). |
|  | *Harmonia* | *axyridis* | 1 | Alyokhin, A., Donahue, C., Majka, C., Chandler, D.S., Hanley, G., Molengraaf, T., Beckendorf, E., Hesler, L. Ladybugs of Maine. Maine Agricultural and Forest Experiment Station. (2011). |
| Curculionidae | *Hypera* | *nigrirostris* | 1 | Titus, E.G. Genera *Hypera* and *Phytonomus* in America, North of Mexico. Annals of the Entomological Society of America. 4(4) 383-473 (1911). |
| Elateridae | *Agriotes* | *sp.* | 1 | Johnson, P.J., 58. Elateridae Leach 1815. in *American Beetles Volume 2 Polyphaga: Scarabaeoidea through Curculionoidea* (eds. Arnett, R.H., Thomas, M.C., Skelley P.E., Frank, J.H.) 617-691 (CRC Press, 2002). |
| Lampyridae | *Ellychnia* | *corrusca* | 1 | Lloyd, J.E. 62. Lampyridae Latreille 1817. in *American Beetles Volume 2 Polyphaga: Scarabaeoidea through Curculionoidea* (eds. Arnett, R.H., Thomas, M.C., Skelley P.E., Frank, J.H.) 187-196 (CRC Press, 2002). |
| Nitidulidae | *Fabogethes* | *nigrescens* | 2 | Audisio, P., Cline, A.R., De Biase, A., Antonini, G., Mancini, E., Trizzino, M., Costantini, L., Strika, S., Lamanna, F., Cerretti, P. Preliminary Re-examination of Genus-Level Taxonomy of the Pollen Beetle Subfamily Meligethinae(Coleoptera: Nitidulidae). *Acta Entomologica Musei Nationalis Pragae*. 49(2) 341-504 (2009) |
| Pyrochroidae | *Pedilus* | *canaliclulatus* | 1 | Young, D.K. Bionomics, Phylogeny, and Systematics of the North American Species of *Pedilus* Fischer (Coleoptera: Pyrochroidae). *Thesis Michigan State University*. (1981). |
| Scarabaeidae | *Valgus* | *hemipterus* | 1 | Ratcliffe, B.C., Jameson, M.L., Smith, A.B.T. 34. Scarabaeidae Latrielle 1802. in *American Beetles Volume 2 Polyphaga: Scarabaeoidea through Curculionoidea* (eds. Arnett, R.H., Thomas, M.C., Skelley P.E., Frank, J.H.) 39-81 (CRC Press, 2002). |
| **Order: Hemiptera** | | | | |
| Berytidae | *Neoneides* | *muticus* | 2 | McAtee, W.L. Key to the Nearctic Genera and Species of Berytidae (Heteroptera). *Journal of the New York Entomological Society*. 27, 79-92 (1919). |
| Membracidae | *Entylia* | *carinata* | 1 | Kopp, D.D., Yonke, T.R. The Treehoppers of Missouri: Part 2. Subfamily Smiliinae; Tribes Acutalini, Ceresini, and Polyglyptini (Homoptera: Membracidae). Journal of the Kansas Entomological Society. 46(2) 233-276 (1973). |
| Miridae | *Lygus* | *lineolaris* | 3 | Schwartz, M.D., Foottit, R.G. Revision of the Nearctic Species of the Genus *Lygus* Hahn With a Review of the Palaearctic Species (Heteroptera: Miridae). (Associated Publishers, 1998). |
| Nabidae | *Hoplistoscelis* | *pallescens* | 1 | Kerzhner I.M., Henry T.J. Three New Species, Notes and New Records of Poorly Known Species, and an Updated Checklist for the North American Nabidae (Hemiptera: Heteroptera). *Proceedings of the Entomological Society of Washington*. 110(4) 988-1011 (2008). |
| Pentatomidae | *Cosmopepla* | *lintneriana* | 1 | McDonald, F.J.D. Revision of *Cosmopepla* Stål (Hemiptera: Pentatomidae). *Journal of the New York Entomological Society*. 94(1) 1-15 (1986). |
| Rhopalidae | *Arhyssus* | *lateralis* | 1 | Chopra, N.P. A Revision of the Genus *Arhyssus* Stål. Annals of the Entomological Society of America. 61(3) 629-655 (1968). |
| **Order: Megaloptera** | | | | |
| Sialidae | *Sialis* | *sp.* | 1 | Liu, X., Hayashi, F., Yang, D. Phylogeny of the Family Sialidae (Insecta: Megaloptera) Inferred From Morphological Data, With Implications for Generic Classification and Historical Biogeography. *Cladistics*. 31(1) 18-49 (2015). |
| **Order: Neuroptera** | | | | |
| Chrysopidae |  |  | 3 |  |
| **Order: Lepidoptera** | | | | |
|  |  |  | 2 |  |

**Table S6.** Results of linear models evaluating agricultural landscape simplification as a predictor of three metrics of insect community composition (Shannon’s H Diversity, Species Richness, and Pielou’s Evenness) for bees, hoverflies, and insects overall. Insects were collected off flowers of *Barbarea vulgaris* plants at 14 sites across a gradient of increasing landscape simplification.

| **Response** | **Predictors** | **Estimate (SE)** | **t** | **P** |
| --- | --- | --- | --- | --- |
| Bee Diversity | Landscape Simplification | -0.07(0.09) | -0.81 | 0.44 |
| Bee Richness | Landscape Simplification | -0.51(0.63) | -0.80 | 0.44 |
| Bee Evenness | Landscape | -0.08(0.06) | -1.30 | 0.22 |
|  | Simplification |  |  |  |
| Hoverfly Diversity | Landscape  Simplification | 0.09(0.08) | 1.03 | 0.32 |
| Hoverfly Richness | Landscape  Simplification | 0.44(0.25) | 1.73 | 0.11 |
| Hoverfly Evenness | Landscape  Simplification | 0.04(0.06) | 0.65 | 0.53 |
| All Insect Diversity | Landscape  Simplification | -0.02(0.07) | -0.24 | 0.82 |
| All Insect Richness | Landscape  Simplification | -0.17(0.76) | -0.22 | 0.83 |
| All Insect Evenness | Landscape  Simplification | -0.07(0.07) | -0.24 | 0.82 |

**Table S7.** Pollinator visitation by species/morpho-group identified “on-the-wing” visiting *B. vulgaris* phytometer and resident plants.

| **Species/Morpho-group** | **Number of Visits Phytometer** | **Number of Visits Resident** |
| --- | --- | --- |
| *Apis mellifera* | 7 | 60 |
| Small Lasioglossum | 450 | 220 |
| Large Lasioglossum | 52 | 5 |
| Small Andrena | 4 | 11 |
| Medium Andrena | 142 | 64 |
| *Melandrena* | 44 | 31 |
| Metallic Green Bee | 91 | 103 |
| *Eristalis* | 142 | 62 |
| *Toxomerus* | 123 | 26 |
| *Hylaeus* | 72 | 0 |
| *Ceratina* | 28 | 0 |
| Other Halictus | 3 | 0 |
| Other Bee | 3 | 4 |
| Other Fly | 37 | 19 |

**Pollinator Visitation Statistical Analysis**

To evaluate pollinator visitation along the landscape gradient, we fit generalized mixed linear models using the package ‘glmmTMB’ which allows for zero inflated negative binomial models^62^. We ran separate models for resident and phytometer plants, as well as for the visitation by bees, hoverflies, and bees in the genus *Lasioglossum*, which accounted for 40% of the total visitation. In both the conditional and zero inflated formulas we included the landscape composition and total flowers and the interaction of the two as predictor variables, with site as a random effect. For phytometer plants, herbivore exclusion was also included as a predictor. In the conditional formulas we also included the log of the observation time as an offset to account for variation in the time each plant was observed due to differences in the length of bloom that restricted the number of observation periods in which the plant was blooming.

**Pollinator Visitation Results**

For phytometer plants, the interaction between landscape simplification and flower number was a significant negative predictor of both the overall bee visitation rate and *Lasioglossum* visitation when evaluated independently. Plants with more flowers had a higher visitation rate, but were also most affected by landscape simplification with visitation rates decreasing as landscapes got simpler (Table 1). While flower number increased visitation rates when a plant received at least one visit, plants with more flowers were less likely to receive any visits overall (Table 1). *Lasioglossum* visitation and total bee visitation were both higher for herbivore excluded plants (Table 1). Hover fly visitation rate was marginally reduced with increasing landscape simplification, but was not affected by herbivore exclusion or flower production (Table 1).

For resident plants, there was a significant negative interaction between landscape simplification and flower number on overall bee visitation (Table 2). However, this pattern did not appear for *Lasioglossum* when evaluated separately or for hover flies (Table 2). Landscape simplification increased both the likelihood that a plant received any hover fly visits as well as the number of visits, while plants with greater flower production received fewer hover fly visits and were less likely to receive any hover fly visits at all (Table 2). For both resident and phytometer plants, bee visitation rate was not predictive of pollinator contribution (the difference in the proportion seed set between bagged and open inflorescences) and therefore was not included in subsequent analyses (Resident plants: F_1,50_ = 0.29, *P* = 0.59; Phytometer Plants: F_1,95_ = 0.03, *P* = 0.85).

***Table S8****. Results of zero inflated negative binomial generalized mixed linear models using the package ‘glmmTMB’ investigating predictors of pollinator visitation to phytometer plants. Landscape Simplification refers to PC1 values from a principal component analysis summarizing pasture, natural, and agricultural land cover at three scales (500, 1000, and 1500m) for each site. Higher values indicate a greater proportion of agriculture in the surrounding landscape and lower values indicate a greater proportion of open (non-forested) natural area and pasture. Lasioglossum visitation is included in addition to overall bee visitation because bees in the genus Lasioglossum were the most abundant visitors, accounting for 40% of all visits. Site was included as a random effect in all models and the log of total observation time was included in the conditional formulas to account for variation in the total time a plant was observed.*

| **Response** | **Formula** | **Predictors** | **Estimate (SE)** | **Z** | **P** |
| --- | --- | --- | --- | --- | --- |
| Bee Visitation | Conditional | Landscape Simplification | 0.17(0.13) | 1.27 | 0.21 |
|  |  | **Flower Number** | **0.76(0.16)** | **4.88** | **1.09e-6** |
|  |  | **Herbivore Exclusion** | **0.54(0.27)** | **1.99** | **0.047** |
|  |  | **Landscape Simplification *Flower Number** | **-0.39(0.10)** | **-4.02** | **5.83e-5** |
|  | Zero-inflation | Landscape Simplification | -0.03(0.23) | -0.12 | 0.90 |
|  |  | **Flower Number** | **-0.97(0.49)** | **-2.00** | **4.60e-2** |
|  |  | Herbivore Exclusion | 0.85(0.82) | 1.04 | 0.30 |
| Lasioglossum | Conditional | Landscape Simplification | -0.31(0.19) | -1.64 | 0.10 |
| Visitation |  | **Flower Number** | **0.53(0.15)** | **3.60** | **3.19e-4** |
|  |  | **Herbivore Exclusion** | **0.68(0.30)** | **2.25** | **2.42e-2** |
|  |  | **Landscape Simplification *Flower Number** | **-0.41(0.14)** | **-2.83** | **4.67e-3** |
|  | Zero-inflation | Landscape Simplification | -0.24(0.26) | -1.15 | 0.36 |
|  |  | **Flower Number** | **-0.73(0.28)** | **-2.58** | **0.01** |
|  |  | Herbivore Exclusion | 0.88(0.58) | 1.52 | 0.13 |
| Hover Fly | Conditional | *Landscape Simplification* | *-0.34(0.19)* | *-1.77* | *0.08* |
| Visitation |  | Flower Number | 0.21(0.20) | 1.07 | 0.28 |
|  |  | Herbivore Exclusion | 0.30(0.33) | 0.92 | 0.36 |
|  |  | Landscape Simplification *Flower Number | 0.02(0.13) | 0.14 | 0.89 |
|  | Zero-inflation | Landscape Simplification | -0.38(0.30) | -1.26 | 0.21 |
|  |  | Flower Number | -2.62(1.58) | -1.56 | 0.12 |
|  |  | Herbivore Exclusion | 0.11(1.36) | 0.08 | 0.94 |

**Table S9.** *Results* *of zero inflated negative binomial generalized mixed linear models using the package ‘glmmTMB’ investigating predictors of pollinator visitation to resident plants. Landscape refers to increasing agricultural land cover in the surrounding landscape. Lasioglossum visitation is included in addition to overall bee visitation because bees in the genus Lasioglossum were the most abundant visitors, accounting for 40% of all visits. Site was included as a random effect in all models and the log of total observation time was included in the conditional formulas to account for variation in the total time a plant was observed.*

| **Response** | **Formula** | **Predictors** | **Estimate (SE)** | **Z** | **P** |
| --- | --- | --- | --- | --- | --- |
| Bee Visitation | Conditional | Landscape Simplification | 0.14(0.10) | 1.44 | 0.15 |
|  |  | Flower Number | 0.10(0.12) | 0.83 | 0.41 |
|  |  | **Landscape Simplification *Flower Number** | **-0.15(0.08)** | **-2.02** | **0.04** |
|  | Zero-inflation | Landscape Simplification | 0.24(0.30) | 0.82 | 0.41 |
|  |  | Flower Number | -0.22(0.36) | -0.63 | 0.53 |
| Lasioglossum | Conditional | Landscape Simplification | 0.08(0.15) | 0.52 | 0.60 |
| Visitation |  | Flower Number | 0.24(0.16) | 1.56 | 0.12 |
|  |  | Landscape Simplification *Flower Number | -0.10(0.09) | -1.05 | 0.30 |
|  | Zero-inflation | Landscape Simplification | 0.28(0.37) | 0.75 | 0.45 |
|  |  | Flower Number | -0.32(0.37) | -0.87 | 0.38 |
| Hover Fly | Conditional | *Landscape Simplification* | *0.91(0.47)* | *1.91* | *0.056* |
| Visitation |  | **Flower Number** | **-0.55(0.28)** | **-2.01** | **0.045** |
|  |  | Landscape Simplification *Flower Number | -0.20(0.15) | -1.33 | 0.18 |
|  | Zero-inflation | *Landscape Simplification* | *1.93(1.10)* | *1.76* | *0.078* |
|  |  | *Flower Number* | *-2.54(1.38)* | *-1.85* | *0.064* |
